# How do phytophagous insects affect phyllosphere fungi? Tracking fungi from milkweed to monarch caterpillar frass reveals communities dominated by fungal yeast

**DOI:** 10.1111/1758-2229.13213

**Published:** 2024-05-13

**Authors:** Ryoko Oono, Vanessa Chou, Mari Irving

**Affiliations:** ^1^ Department of Ecology, Evolution, and Marine Biology University of California Santa Barbara California USA

## Abstract

Since a significant proportion of plant matter is consumed by herbivores, a necessary adaptation for many phyllosphere microbes could be to survive through the guts of herbivores. While many studies explore the gut microbiome of herbivores by surveying the microbiome in their frass, few studies compare the phyllosphere microbiome to the gut microbiome of herbivores. High‐throughput metabarcode sequencing was used to track the fungal community from milkweed (*Asclepias* spp.) leaves to monarch caterpillar frass. The most commonly identified fungal taxa that dominated the caterpillar frass after the consumption of leaves were yeasts, mostly belonging to the Basidiomycota phylum. While most fungal communities underwent significant bottlenecks and some yeast taxa increased in relative abundance, a consistent directional change in community structure was not identified from leaf to caterpillar frass. These results suggest that some phyllosphere fungi, especially diverse yeasts, can survive herbivory, but whether herbivory is a key stage of their life cycle remains uncertain. For exploring phyllosphere fungi and the potential coprophilous lifestyles of endophytic and epiphytic fungi, methods that target yeast and Basidiomycota fungi are recommended.

## Graphical abstract

Fungal diversity was tracked from milkweed (*Asclepias* spp.) leaves to monarch caterpillar (*Danaus plexippus*) frass with metabarcoding to understand potential adaptations of phyllosphere fungi to folivory. The number of fungal species decreased from leaves to frass, with no consistent species increasing in relative abundance, implying a random bottleneck occurs in caterpillar guts. However, Basidiomycota yeasts were notably prevalent in caterpillar frass, suggesting that the yeast growth form could be conducive to surviving caterpillar folivory..

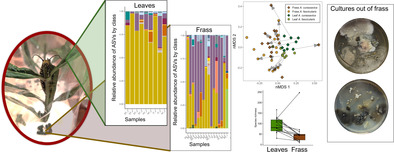

## INTRODUCTION

Fungi in the phyllosphere, including endophytes and epiphytes, represent a functionally and phylogenetically diverse group that is ubiquitously, but to varying degrees, associated with all plants (Morse et al., 2007; Rodriguez et al., 2008, 2009). They have a wide variety of life histories ranging from latent pathogens (Drake‐Schultheis et al., 2020; Granados et al., 2020) to mutualists, the latter capable of enhancing growth (Wang et al., 2016), nutrient acquisition (Chhabra & Dowling, 2017) or stress tolerance in their hosts (West, 1994). Although all phyllosphere microbes may immediately benefit from the symbiotic association by using their host as a habitat, it is not yet clear how they gain fitness benefits or have adapted to reproduce from their host's tissues. Plant hosts are frequently and regularly (at least over evolutionary time scales) consumed by herbivores (e.g., up to 20% of plant material in forests succumb to herbivory (Cyr & Face, 1993), making herbivory a likely fate for most phyllosphere microbes. And yet, it is unclear if phyllosphere fungi are adapted to obligately reproduce from their hosts' tissues and would benefit from any protections from herbivores of their hosts, or are more flexible and capable of surviving the guts of herbivores as coprophiles.

Avoiding herbivory is often assumed to be beneficial for both plants and their symbiotic fungi (Herre et al., 2007). For example, one of the best‐studied fungal endophytes of grasses produces alkaloids that deter herbivores and are adapted for vertical transmission through host seeds (Clay, 1988; Gundel et al., 2018; Schardl et al., 2004). However, in contrast to grass hosts that are infected by the less‐diverse, vertically transmitted endophytes (Class I, sensu Rodriguez et al., 2009), the vast majority of terrestrial plants are infected with multiple lineages of endophytes and epiphytes by horizontal transmission through senescent leaves or leaf surfaces (Davis et al., 2003; Kumar et al., 2017; Petrini, 1986; Saikkonen et al., 1998). This highly diverse community of fungi appears to be largely unspecialised with weak toxicity against herbivores (Bamisile et al., 2018). Still, many phyllosphere fungi, especially endophytes, are hypothesised to reproduce by sporulation from dead senesced leaves (Osorio & Stephan, 1991), suggesting that herbivores preempt phyllosphere symbionts the opportunity to reproduce. While this hypothesised life cycle suggests a fitness alignment between plant hosts and symbionts, the effect of herbivory on plant symbiont life history has yet to be thoroughly investigated. A fitness alignment has great implications for the evolutionary trajectory of the plant microbiome and merits further investigation.

While we often study the phyllosphere microbiome for its ecological relationship with the plant host, we may be overlooking a key life stage of many of these microbes if they are also consistently present in the gut microbiomes of herbivores. The influence of herbivory on the life history of plant symbionts has not been extensively studied, except for the alkaloids evolved in grass endophytes in response to herbivory (Clay, 1988; Gundel et al., 2018; Schardl et al., 2004). Microbial symbionts of herbivores, on the other hand, are often studied for their significant roles in aiding digestion, such as by detoxifying plant compounds (Francoeur et al., 2020), breaking down cellulose or lignin (Scully et al., 2013; Watanabe & Tokuda, 2010), facilitating the development of healthy gut microbiota (Shikano et al., 2017; Shao, 2017), and mediating interspecific and intraspecific host communication (Di Salvo, 2019; Hammer, 2015). It remains untested whether phyllosphere microbes could be propagated and selected, not only by plant hosts but also by plant herbivores. It is certainly possible that some plant microbes are not necessarily adapted to reproduce on senescent leaves, but instead are beneficial to the health of herbivores and survive to reproduce post‐herbivory with a coprophilous lifestyle.

In this study, we use high‐throughput amplicon sequencing to track changes in the fungal community from milkweed leaves to monarch caterpillar frass. Milkweeds and monarchs are some of the best‐studied systems in evolution and ecology, but little is known regarding the microbiome inhabiting either species. Hansen and Enders (2022) explored the bacterial microbiome of milkweeds but the fungal microbiome has yet to be thoroughly surveyed (see Fouda et al., 2015; Holbrook, 2017). The milkweed genus (*Asclepias*, Family Apocynaceae) provides a unique host system to track the phyllosphere microbiome through herbivore consumption as they are the exclusive food source for the larvae of the monarch butterfly (*Danaus plexippus*). The microbial diversity of milkweeds could also be a specialised community adapted to the constitutive and inducible array of plant defence compounds, most notably cardenolides, which have both herbicidal and fungicidal properties (Akhtar & Isman, 2003; Green et al., 2011; Petschenka et al., 2011; Sachdev‐Gupta et al., 1993). We investigated which fungal taxa dominated the caterpillar frass after the consumption of leaves and identified any candidate fungal taxa that may be adapted to survive caterpillar herbivory for future studies.

## EXPERIMENTAL PROCEDURES

### Plant and frass collection

Stems from two prevalent milkweed species were collected from Santa Barbara County in July 2018. Stem cuttings of *Asclepias fascicularis*, a native of California, were collected from the Cheadle Center for Biodiversity and Ecological Restoration site while stem cuttings of *A. curassavica* (Mexican butterfly weed, sold most commonly at California nurseries and native to Mexico) came from the University of California Santa Barbara Greenhouse grounds (1.5 km apart). Monarch eggs and juvenile caterpillars all came from the *A. curassavica* samples. Although the milkweeds are growing outdoors with no immediate canopies, they are located among buildings with non‐native plants nearby. The milkweeds have been growing in these areas for several decades, possibly allowing any specialised phyllosphere fungi to have had time to increase in population locally. The two milkweed species differ considerably in cardenolide concentrations (Ladner & Altizer, 2005), and are exposed possibly to different fungal inoculums due to different locations. While we report differences between the phyllosphere microbiomes of the two milkweed populations, the primary focus of this study is the change in microbial communities through the gut passage of caterpillars.

**FIGURE 1:**
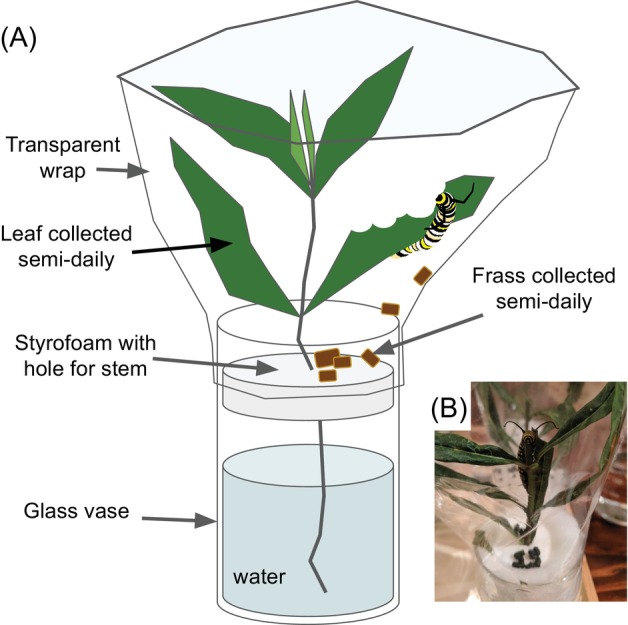
Leaf and frass collection set‐up. (A) Diagram of set‐up allowing collection of caterpillar frass and opposite leaf tissues in pairwise fashion. Freshly cut stems with at least four mature leaves were often provided every day. (B) Photograph of one of the caterpillar enclosures.

Milkweed stems were kept in water in individual vases and one caterpillar was transferred to one stem with at least four leaves. The milkweeds were not surface‐sterilised. Each stem was wrapped with clear plastic wraps to prevent the caterpillars from moving off plants and to collect all of the frass (Figure 1). The milkweed stems were inserted into styrofoam bases that collected any caterpillar droppings and the droppings were collected daily into microcentrifuge tubes with sterilised forceps for each caterpillar. Since milkweed leaves grow in opposite formations, after caterpillars had eaten one leaf, we collected the opposite uneaten leaf and recorded its date. Frass and leaves were saved in −20°C. The sampling for DNA extraction took place between 3 July 2018 and 5 July 2018.

### Amplicon sequencing

DNA from plant tissues and frass were extracted following a modified CTAB protocol (Branco et al., 2015). The internal transcribed spacer (ITS) region 1 was amplified using the ITS1F‐KYO1 and ITS2‐KYO1 (Toju et al., 2012) primers modified with Illumina overhang adaptors. Barcodes were added and pooled samples were sequenced with paired‐end sequencing (2 × 250 bp) on an Illumina MiSeq sequencer at the California Nanosystems Institute at the University of California Santa Barbara (Santa Barbara, CA) with a 15% spike‐in of PhiX. A detailed description of the preparation of the library can be found in Appendix S1. In addition, purified PCR4‐TOPO vectors (Invitrogen, Carlsbad, CA, USA) containing the targeted ITS1 region from two fungal species (one Basidiomycota and one Ascomycota), were used as positive controls to assess ‘sample bleeding’ or ‘cross‐talking’ (Mitra et al., 2015; Sinha et al., 2017). Demultiplexed, raw sequence reads are available in the NCBI SRA under BioProject accession PRJNA660094.

### Bioinformatics pipeline and statistical analyses

Sequences were filtered and denoised into amplicon sequence variants (ASVs; Callahan et al., 2017) using a custom *dada* pipeline (Appendix S2). Fungal ASVs were taxonomically filtered using MEGAN (Huson et al., 2007) and also manually checked with BLAST (Altschul et al., 1990). Common ASVs with 99%–100% sequence similarity to voucher specimens in GenBank are referenced at their genus level as most ASVs cannot be confidently identified to species. The Bray–Curtis dissimilarities of the total endophyte communities were calculated after normalisation with a cumulative sum scaling technique using the *metagenomeSeq* package (Paulson, Stine, et al., 2013; Paulson, Pop, & Bravo, 2013) and ‘vegdist’ command from the package *vegan* v2.5‐4 (Oksanen et al., 2016). The samples were sequenced across two separate MiSeq libraries with some of the same samples sequenced twice (Appendix S3). We combined duplicate samples after we determined that the two libraries were not significantly different (*R* ^2^ = 0.007, *p* = 0.87) based on permutational multivariate analysis of variance (PERMANOVA) using the ‘adonis’ command from the *vegan* package (Oksanen et al., 2016).

Using the Bray–Curtis dissimilarity matrices, we constructed non‐metric multidimensional scaling (nMDS) plots of the amplicon community data for total fungal ASVs. We used a BIOBIO step‐wise algorithm modified in *vegan* that explores the taxonomic variables that best correlate to the community dissimilarity patterns (see Appendix S4 for script). We assessed whether the fungal communities were different between milkweed species and between frasses from the consumption of different milkweed species with PERMANOVA. We also used the ‘multipatt’ function in the *indicspecies* package (De Cáceres & Legendre, 2009) to identify fungal species that are significantly associated with either (1) leaves, (2) frass or (3) leaf and frass of one milkweed species and not the other. We considered all leaf samples to be independent samples since they were fresh cuttings. However, some frass samples come from the same caterpillar individuals sampled from different days. Any time we considered a frass sample as an independent variable, we merged the frass samples by the caterpillar or blocked by the caterpillar. We tested for indicator species using a much smaller subset of ASVs based on average read abundance (the union set for the top 10 read‐abundant ASVs per group) and the 14 ASVs that highly correlated with community dissimilarity using the BIOBIO algorithm. The total number of ASVs tested with the indicator species analysis was 25 (see Appendix S5 for ASV identification).

### Culturing fungi from frass samples

To test if fungi from frass samples were viable, frozen frass samples were reconstituted on 2% malt extract or Sabouraud's media. A random set of cultures were sequenced for ITS1, ITS2 or LSU, depending on amplification success. A second set of frass was acquired from individual caterpillars as they consumed plants from the field. Caterpillars were being observed and frass were collected with sterile forceps immediately after exiting the bodies of the caterpillar. The frass was immediately smeared on 2% ME and Sabouraud's plates. During the collection and culturing, which all took less than 15 s per plate, we had additional media plates exposed to the environment for 1 h as negative controls. We poked the negative control plates with sterile forceps and added approximately 20 μL of sterile water to help attract any spores in the surrounding air.

## RESULTS

### Amplicon sequencing of fungi

The library produced 2.49 M reads for 99 samples, including two positive controls. The reads were denoised to 2690 ASVs, 2032 of which were assigned to Fungi based on default MEGAN criteria and manual BLAST filtering. The rest of the ASVs were categorised as ‘No Hits’ (275/2690, 10%), or bacteria (383/2690, 14%). The two positive controls produced either two or three ASVs total that were assigned to the correct taxa, suggesting that ‘bleeding’ or ‘barcode‐skipping’ was insignificant in these libraries.

**FIGURE 2:**
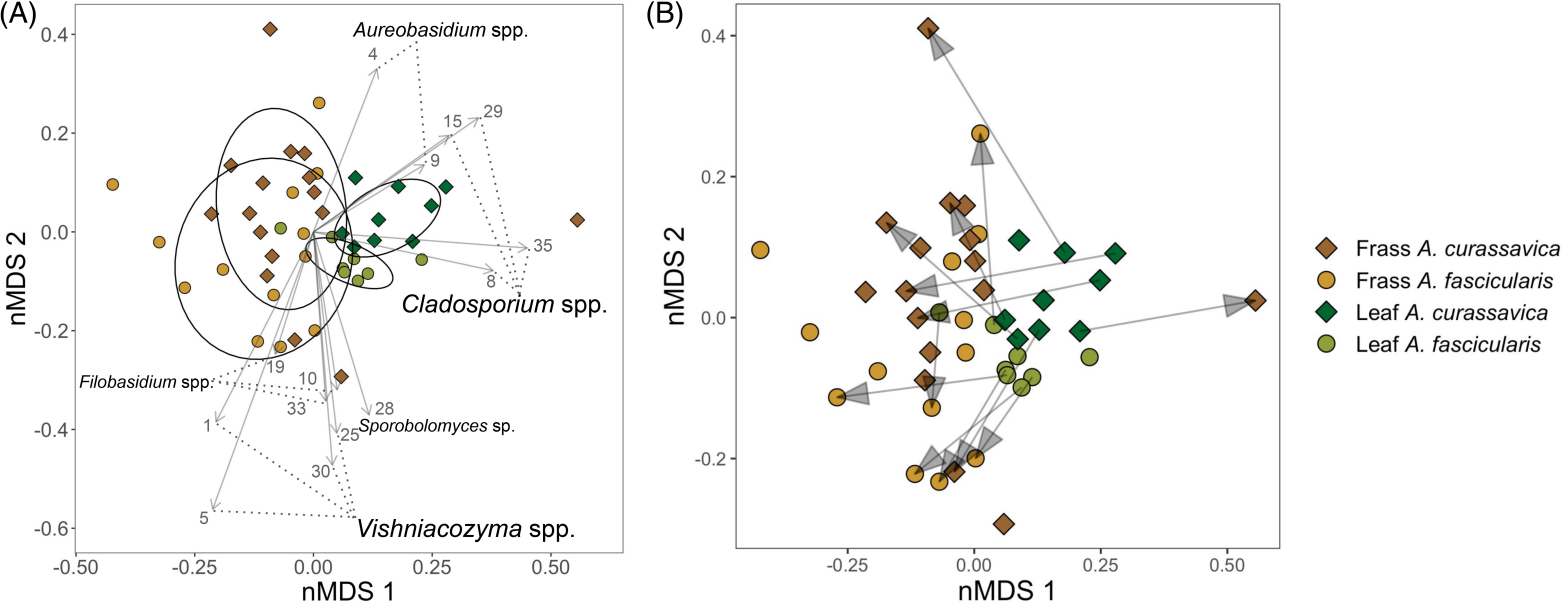
Representation of the communities of phyllosphere fungi from the leaves of two milkweed species and the frass produced from the consumption of these two species by monarch caterpillars with non‐metric multidimensional scaling (nMDS). (A) Fungal communities are distinct between leaves and frass. Ellipses represent one standard deviation. Arrows represent 14 amplicon sequence variants (ASVs) whose read abundance is significantly associated with the representation on the nMDS using BIOBIO. The ASV numbers are indicated by the vectors and their top BLAST hits can be found in Appendix S6. (B) Arrows designate paired samples showing how the fungal communities change from plant to frass. Stress = 0.181.

Fungal communities from the leaves, including endophytes and epiphytes, of the two milkweed species were significantly different (PERMANOVA *R* ^2^ = 0.184, *p* < 0.001, Figure 2). The fungal communities of the frasses (merged by caterpillar individuals) originating from the consumption of different milkweed species were also different from each other (PERMANOVA *R* ^2^ = 0.138, *p* = 0.038), but not as different as the fungal communities of the leaves. Although the fungal communities in the frass for the nMDS analysis are sometimes derived from the same caterpillar individuals, the variation or beta‐dispersion of the communities was greater than that from the leaves that those caterpillars were consuming. The average distance from the median of a frass group was 0.48 to 0.49 on the nMD scale, but 0.35–0.37 for the leaf groups. The turnover among fungal communities from the frass (i.e., beta diversity) was, hence, greater than for fungal communities from the leaves. As can be seen in Figure 2B, where we indicate fungal community pairs with vectors, the direction (vector angles) and extent (vector scales) of the change of the fungal communities from the phyllosphere to the frass were highly variable.

**FIGURE 3:**
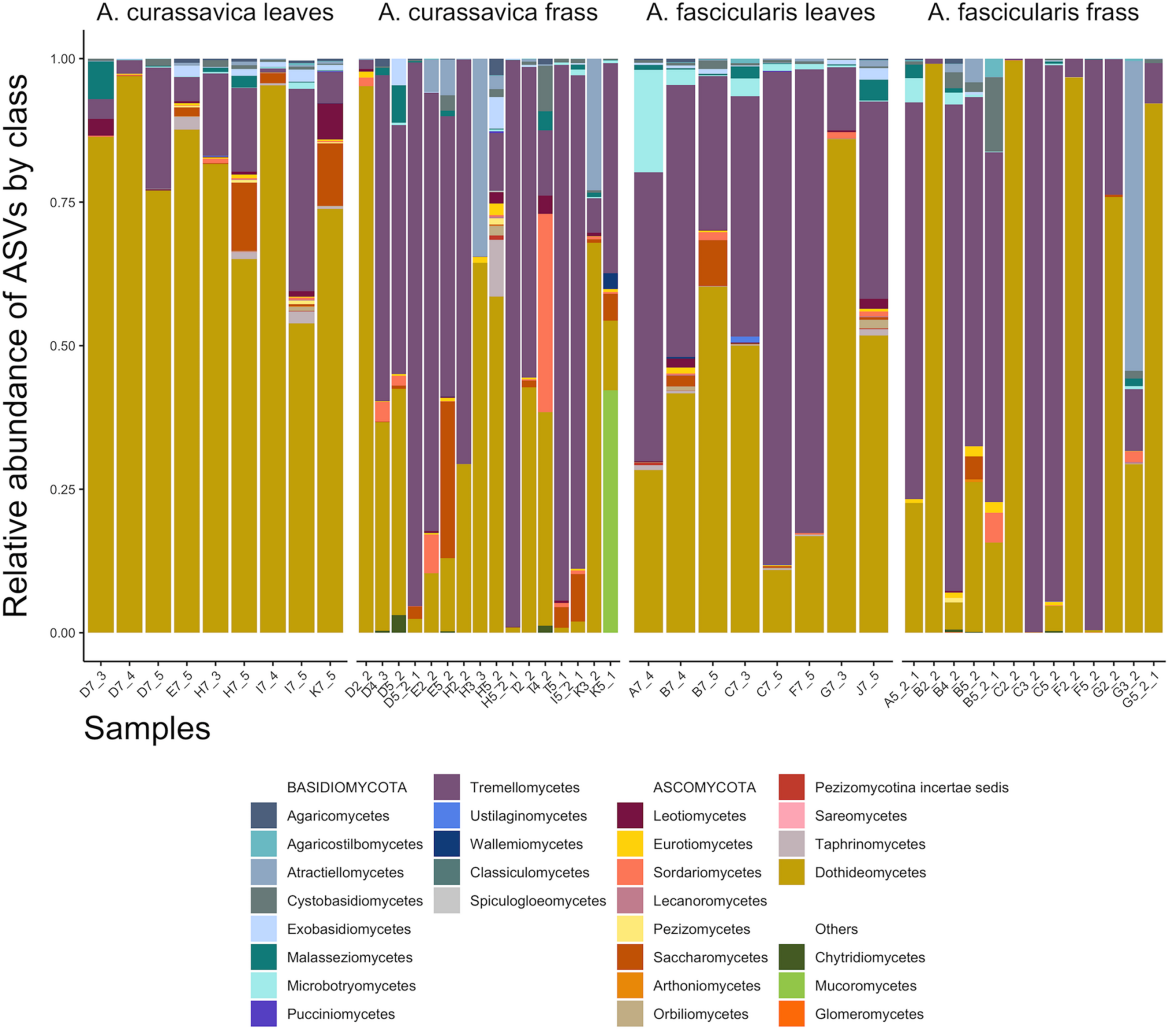
Relative abundance of fungal amplicon sequence variants (ASVs) by class. Samples were rarefied to 1728 reads. X‐axes are individual leaf or frass samples (letters indicate caterpillar individuals, that is, D7_3 = leaves eaten by Caterpillar D on 3 July or D2_2 = second frass collected from Caterpillar D on 2 July).

**FIGURE 4:**
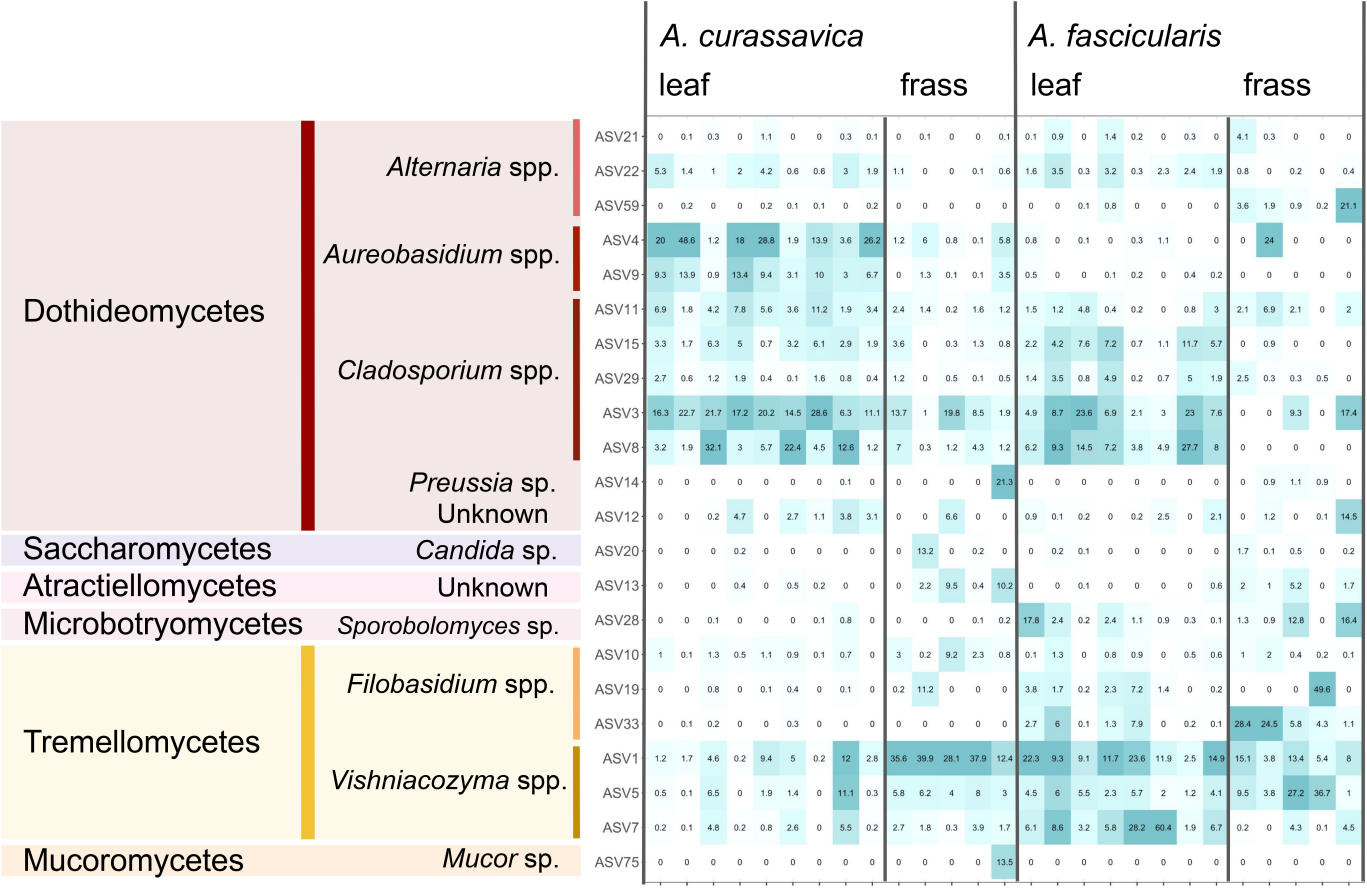
Top 10 amplicon sequence variant (ASVs) with the highest average read abundance from each of the four treatment groups. Samples from the same caterpillars from different dates were merged. Tiles are shaded based on their prevalence within a single sample. Hence, a tile is dark‐coloured if it is dominant in that sample relative to other ASVs, not relative to other ASVs in other samples. The top BLAST hits for each ASV are noted in Appendix S6.

We found 12 ASVs to be highly correlated with the community profiling using nMDS (Figure 2A); four *Cladosporium* spp. Link (Dothideomycetes), four *Vishniacozyma* spp. Liu, Bai, Groenewald & Boekhout (Tremellomycetes), two *Aureobasidium* spp. Viala & Boyer (Dothideomycetes), three *Filobasidium* spp. Olive (Tremellomycetes) and one *Sporobolomyces* sp. Kluyver & Niel (Microbotryomycetes). Fungal genera that were among the top 10 most abundant in each group (Figures 3 and 4; Appendix S5) included *Cladosporium*, *Alternaria* Nees, *Aureobasidium* and *Preussia* Fuckel in the Dothideomycetes (36.5% of reads), *Vishniacozyma* and *Filobasidium* in the Tremellomycetes (17.7% of reads), as well as *Candida* Berkhout (Saccharomycetes), *Sporobolomyces* (Microbotryomycetes), *Mucor* Fresen. (Mucoromycetes) and an unknown genus in *Atractiellomycetes* (Pucciniomycotina, Basidiomycota). The top BLAST hits of notable ASVs can be found in Appendix S6.

Based on the indicator species analysis, four ASVs were significantly associated with frass and one was significantly associated with *A. fascicularis* (*p <* 0.05 after correction for multiple testing, Appendix S5). The four ASVs strongly associated with frass, but not leaves (although they are all found in some leaf samples), included two *Vishniacozyma* spp. (ASV1 and ASV5), a *Filobasidium* (ASV10), and an unknown Atractiellomycetes (ASV13)—all Basidiomycota. One ASV (ASV33), a *Filosbasidium* sp., was associated with *A. fascicularis* leaves and frass but not with samples from *A. curassavica*. We also note that one ASV (ASV35) identified as a *Cladosporium* sp., a filamentous Ascomycota, was marginally associated with leaves of both milkweed species (*p =* 0.013, adjusted *p* = 0.053), but not with either of the frass. This ASV was not one of the most abundant ones in terms of read numbers but was indicated as significantly correlated with the nMDS structure (Figure 2A).

**FIGURE 5:**
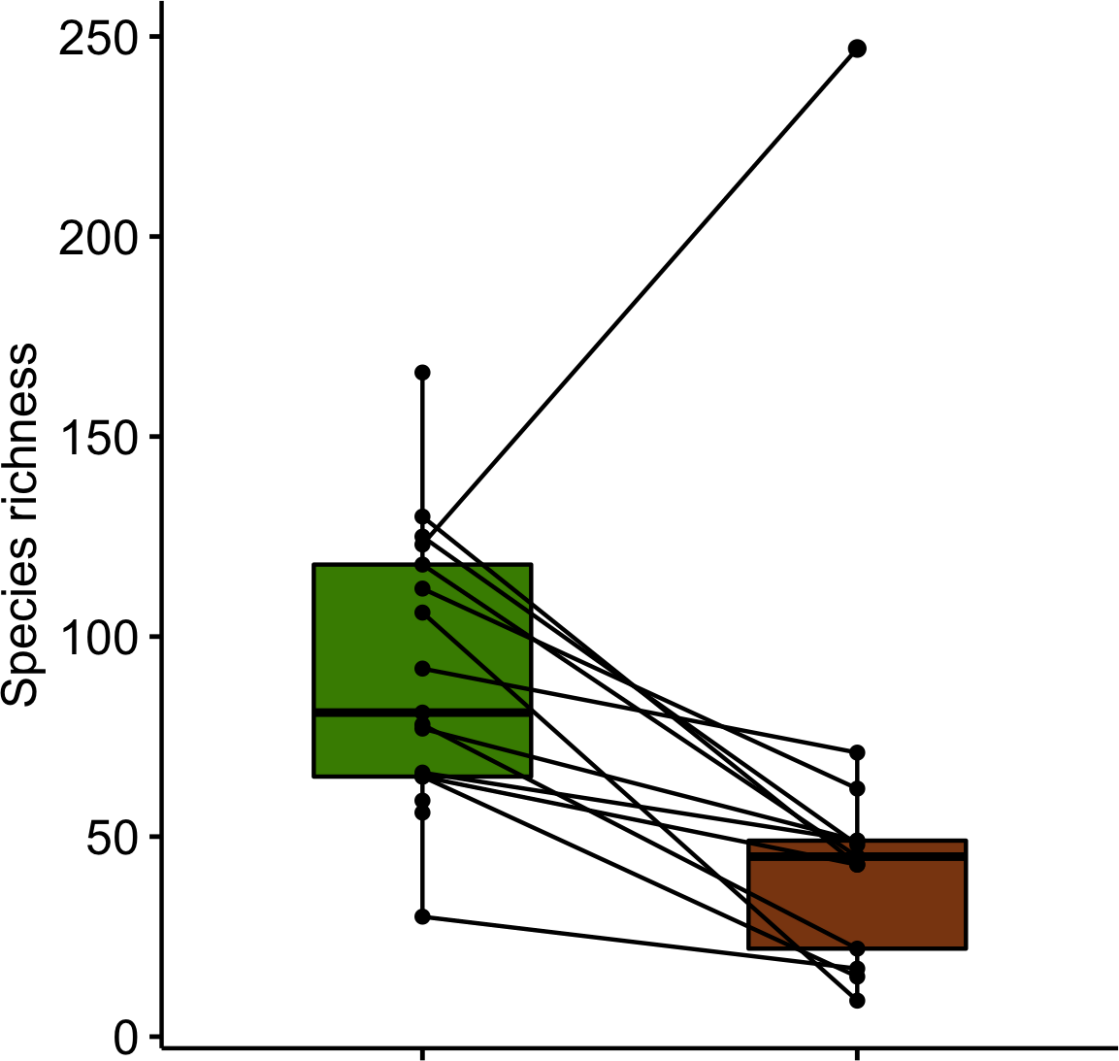
The number of amplicon sequence variants from rarefied samples (1728 reads per sample) in plant and frass samples. Samples for which pairings were sequenced have a line drawn between them. Some leaf samples do not have a corresponding frass sample.

The average ASV richness of rarefied samples was less in frass (68.5 ASVs ±33) compared to the leaves (91.1 ± 34), although this was not statistically significant with a *t*‐test. The test was significant (*t* = −2.76, df = 22.84, *p*‐value = 0.011) after the removal of one outlier from the frass group (see Figure 5; Appendix S7). We note that a non‐paired *t‐*test is also more conservative than a paired *t‐*test.

### Culturing fungi from frass samples

Although culturing frass samples that were once frozen may have limited recovery of some fungal taxa, we isolated a morphologically diverse set of fungal colonies from frass samples on Sabouraud's media and 2% malt extract. Some of these were sequenced (most did not amplify with our primers) and some returned as yeast, such as *Candida*. We also compared fungal growth from plates with fresh smeared frass and plates that were exposed to the environment and found significantly more growth from the plates with fresh smeared frass (Appendix S8).

## DISCUSSION

Despite significant proportions of foliage consumption by herbivores (Cebrian, 1999; Cyr & Face, 1993; Hollinger, 1986; Zvereva et al., 2012), the impact that herbivory has on the life history of phyllosphere fungi remains uncertain. Whether phyllosphere symbionts are harmed or benefit from herbivory has significant implications for their evolutionary trajectory and relationship with their host plant. This study sought to determine if any members of the phyllosphere fungi, including endophytes and epiphytes, of two milkweed species might consistently survive or increase in abundance post‐digestion by the holometabolous monarch caterpillar. We observed that leaf fungal communities undergo a bottleneck by caterpillar herbivory based on significantly decreased species richness from leaf to frass. However, there appeared to be no consistency in the directionality of the changing community composition. The fungal communities from the frass of caterpillars feeding on different milkweed species were not as distinct from each other as those from the respective leaves.

The most prevalent fungal taxa present in the milkweed phyllosphere (*Cladosporium*, *Alternaria*, *Vishniacozyma*, *Sporobolomyces*, *Aureobasidium*) are also abundantly found in diverse plant lineages (Appendix S6, Félix et al., 2020; Last, 1955; Sibanda et al., 2018; Wang et al., 2007). Hence, despite their unique toxicity, milkweed leaves do not appear to house specialist phyllosphere fungal taxa (at least at a coarse genus level) that might be specifically adapted to cardenolides. Phyllosphere microbes on plants with a high likelihood of herbivory may be generalists who can reproduce on faeces or soil rather than from senesced litter of specific plant species. Endophytes of broad leaves are also more generalist compared to those found on evergreen leaves (Apigo & Oono, 2021; Vincent et al., 2016 ), such as pines (Sarver et al., 2022), which break down less easily. We note that this study only examined two of over 200 species of *Asclepias* and that specialist phyllosphere fungi may exist in other milkweed species or environments.

Four of the five most common fungi in both plant and frass were yeasts: *Vishniacozyma*, *Filobasidum*, *Sporoblomyces* and *Aureobasidium*. These genera have been reported from a wide range of hosts, including from extreme environments such as arctic glaciers and the deep sea (Buzzini et al., 2018; Ogaki et al., 2020; Singh et al., 2013; Tsuji et al., 2019). Caterpillar guts are also an extreme environment for microbes due to their high alkalinity and undesirable physiological attributes, such as a simple tube morphology that prevents microbe housing structures (Appel, 2017; Johnson & Felton, 1996). Interestingly, many of the fungal yeasts are also common in the phylloplane (Haelewaters et al., 2021) and some are considered mutualists of plants by producing antimicrobial and growth‐promoting metabolites (Hamayun et al., 2009; Wang et al., 2007). The majority of the closest BLAST matches to sequences in this study also came from the phyllosphere (Appendix S6).

*Cladosporium* was another fungus abundantly found in the milkweed phyllosphere; although they are not yeasts, they are commonly found endophytes in many species such as birches, oaks, strawberries and soybeans, easily spread through the air and have varying functional roles from mutualist to pathogen (Harris et al., 2000; Wang et al., 2007, 2013). One *Cladosporium* ASV abundance declined from plants to frass, further suggesting that a yeast life history may allow better survival for phyllosphere microbes from herbivory rather than a filamentous morphological form.

Upon performing an indicator species analysis, we found only three fungal taxa (*Vishniacozyma* spp., *Filobasidium* sp., and an unknown Atractiellomycetes) increased in relative dominance from plant to frass material (Appendix S5). Although these ASVs were found in the sampled milkweed leaves, their low abundance in the leaves and high abundance in the frass may be due to their origins from non‐leaf tissues, such as stems and flower buds, which the caterpillars also devour but which we did not sample. We also note that some microbes identified in this study from caterpillar frass may have non‐milkweed origins since these caterpillars, albeit from early instars, were collected from outside and may have been exposed to soil microbes (Hannula et al., 2019).

Interestingly, although statistically insignificant, caterpillar frass contained 39 ASVs with close affiliation to Chytridiomycota, which were not found in milkweed leaves (and are rarely found in plants). The majority of these ASVs could not be further narrowed taxonomically because their closest BLAST hits were often ‘uncultured Chytridiomycota clone’ (Monchy et al., 2011) or to a broad subgroup of Chytridiomycota, such as Rhizophydiales (Van den Wyngaert et al., 2022, see Appendix S6 for BLAST hits of a subset of these ASVs). Given the paucity of Chytridiomycota sequences in GenBank, the taxonomic similarities of many of our sequences to any references were often low (e.g., 80% sequence similarity) and the query coverages were even lower (e.g., <20%), and cannot be verified at this time. However, this is still noteworthy as an unusual phylum to be identified from processed plant matter. We leave the possibility that chytrids could be resident microbes of the caterpillars, acquired from non‐plant sources. Chytridiomycosis (caused by *Batrachochytrium dendrobatidis*; Rhizophydiales) is an emerging infectious disease in amphibians but has not been previously reported in any Lepidopteran species. These chytrids could either be additional pathogens of monarch hosts or even potential enemies of monarch parasites, such as protozoan *Ophryocystis elektroscirrha* (Bradley & Altizer, 2005) or tachinid fly *Lespesia archippivora* (Oberhauser et al., 2017).

Caterpillars are suggested to lack a resident microbiome and possess unfavourable digestive physiology for the growth of most microbes (Hammer et al., 2017), but they still consume many phyllosphere microbes and potentially aid a coprophilous lifestyle. Based on non‐culturing methods, we found overlapping taxonomic composition between the phyllosphere and frass communities and several fungal lineages consistently increasing in abundance in caterpillar frass across multiple caterpillar individuals. We have also shown that more viable fungal cultures are found in caterpillar frass relative to the open air. While the identification of some yeast cultures from frass samples only suggests that the frass environment is amenable to yeast growth and does not prove that endophytic yeasts survive the guts of caterpillars, this study provides evidence for the possibility that fungal communities, especially yeasts, are selected by herbivory.

The dominance of diverse yeasts in monarch and milkweed ecologies is notable, but their ecological significance remains uncertain. Some predators and parasites of milkweeds have evolved similar genetic mechanisms to tolerate cardiac glycosides (Groen & Whiteman, 2021), suggesting microbes may also have convergent evolutionary traits to inhabit the milkweed phyllosphere. Investigating yeast tolerance against cardenolides or interactions with milkweed pathogens and monarch parasites could provide fruitful future discoveries that may aid in monarch conservation. Some of these endophytes may also aid in plant growth (Fouda et al., 2015), which may help in the restoration efforts of milkweed populations. Furthermore, the dominance of yeasts, in particular Basidiomycota yeasts, from this study suggests future culture‐based studies should carefully consider multiple growth media that can cultivate both filamentous fungi and yeast.

## AUTHOR CONTRIBUTIONS

**Ryoko Oono:** Conceptualization (lead); data curation (equal); formal analysis (lead); funding acquisition (lead); investigation (equal); methodology (equal); project administration (lead); supervision (lead); validation (lead); visualization (lead); writing – original draft (lead); writing – review and editing (lead). **Vanessa Chou:** Data curation (equal); investigation (equal); methodology (equal); writing – review and editing (supporting). **Mari Irving:** Data curation (supporting); investigation (supporting); validation (supporting); writing – original draft (supporting); writing – review and editing (supporting).

## CONFLICT OF INTEREST STATEMENT

Authors have no conflict of interest to declare.

## Supporting information


**APPENDIX S1.** Detailed methods for culture‐independent amplicon sequencing and bioinformatics.

**APPENDIX S2.** R script for dada pipeline.

**APPENDIX S3.** Metadata file for samples used in the analysis.

**APPENDIX S4.** R script for all community analyses.

**APPENDIX S5.** Results of indicator species analysis.

**APPENDIX S6.** Top BLAST hits of ASVs mentioned in this study.

**APPENDIX S7.** Observed ASV richness from the rarefied community (1728 reads per sample).

**APPENDIX S8.** Cultured fungi from caterpillar frass immediately collected from caterpillars.

## Data Availability

All data are contained within the article. Sequencing data are available in NCBI SRA under BioProject accession PRJNA660094. Any other detailed methods, data and scripts are in Supporting Information.
