# Supplementary material for: How do phytophagous insects affect phyllosphere fungi? Tracking fungi from milkweed to monarch caterpillar frass reveals communities dominated by fungal yeast
Source: Environ Microbiol Rep. 2024 May 13;16(3):e13213. doi: 10.1111/1758-2229.13213 (PMC11089944; doi:10.1111/1758-2229.13213)
Supplement: Supplementary file 1 — APPENDIX S1. Detailed methods for culture‐independent amplicon sequencing and bioinformatics. [file EMI4-16-e13213-s002.pdf]

Appendix S1. Detailed methods for culture-independent amplicon sequencing and bioinformatics

DNA from whole plant tissues or caterpillar frass were extracted following a modified CTAB protocol (Branco *et al.*, 2015; Oono *et al.*, 2017; http://1000.fungalgenomes.org/home/protocols/). The ITS1 region was amplified using the ITS1F-KYO1 and ITS2-KYO1(Toju *et al.*, 2012) primers modified with Illumina overhang adaptors. The first stage of amplification was carried out in a total volume of 25 µl using 10 ng of DNA, 0.1 µM of BSA, 2.5 µl of 10x PCR buffer containing 15 mM $MgCl_2$, 200 µM of each dNTP, 0.75 units of Choice-Taq DNA polymerase (Denville Scientific Inc, Holliston, MA, USA), and 0.5 µM of each primer. The PCR conditions were 3 min at 95 °C, followed by 35 cycles of 30 s at 95 °C, 30 s at 47 °C, 30 s at 72 °C, and a final elongation of 5 min at 72 °C. Duplicate PCR reactions were run per sample and 5 µl of pooled PCR products were used as template in the second PCR using custom barcode primers designed after Illumina's Nextera XT Index Kit v2 (i5 and i7 indexes). The second PCR consisted of 3 min at 95 °C, followed by 10 cycles of 30 s at 95 °C, 30 s at 55 °C, 30 s at 72 °C, and a final elongation for 5 min at 72 °C. Amplicons were purified with Agencourt AMPure XP SPRI magnetic beads (Beckman Coulter, Brea, CA, USA) using a 1:1 ratio, and normalized to 4 nM.

Two cloned sequences, one Ascomycota and one Basidiomycota, were also amplified as positive controls. The sequences are deposited at MG840195 and MG840196.

Demultiplexed paired-end reads were assembled using the DADA2 pipeline (version 1.16) (Callahan *et al.*, 2016). Non-biological sequences, such as adapters and primers, were removed prior to assembly. Reads were filtered using standard parameters: truncation quality score equal to 2 (truncQ=2) and reads with maximum expected errors greater than 2 were discarded (maxEE=2). Forward and reverse reads were merged to obtain full sequences by aligning the forward reads with the reverse-complement of the corresponding reverse reads, and then constructing the merged "contig" sequences. Merged sequences are only output if forward and reverse sequences overlap by a minimum of 12 base pairs and are identical in the overlap region. Chimeric sequences were identified if they could be reconstructed by combining a left and right segment from two more-abundant sequences. The resulting amplicon sequence variant (ASV) table was used in a BLAST (Altschul *et al.*, 1990) search against the entire GenBank nucleotide database excluding sequences that originated from environmental sampling (ftp://ftp.ncbi.nlm.nih.gov/blast/db/nt*, downloaded on January 8, 2016) and outputs were parsed in MEGAN 4 (Huson *et al.*, 2011) for taxonomic assignment.

In total, 2,690 ASVs were parsed in MEGAN 4 for taxonomic assignment (minimum score threshold of 170, minimum hit support of 1 read, max percent of best score 5 %). ASVs classified as 'No hits' (275) typically had lengths <100 bps and were all excluded from the final ASV table. ASVs classified in Viridiplantae (47) were excluded from the final ASV table. All

ASVs under ‘Not assigned’ (275) were manually checked for potential classification within Fungi with BLAST but no sequences were included in final ASV table. All ASVs classified to the Ophisthokonta were also manually checked. In the end, we included all 59 sequences assigned to Ophisthokonta to the final ASV table. The final number of ASVs came to 2,032 with 1,973 correctly assigned to Fungi by MEGAN.

The 2,032 fungal ASVs were exported from MEGAN to construct the final fungi-only ASV abundance table using an R script.

Altschul, S.F., Gish, W., Miller, W., Myers, E.W., and Lipman, D.J. (1990) Basic local alignment search tool. *J Mol Biol* **215**: 403–410.

Branco, S., Gladieux, P., Ellison, C.E., Kuo, A., LaButti, K., Lipzen, A., et al. (2015) Genetic isolation between two recently diverged populations of a symbiotic fungus. *Mol Ecol* **24**: 2747–2758.

Callahan, B.J., McMurdie, P.J., Rosen, M.J., Han, A.W., Johnson, A.J.A., and Holmes, S.P. (2016) DADA2: High-resolution sample inference from Illumina amplicon data. *Nat Methods* **13**: 581–583.

Huson, D.H., Mitra, S., Ruscheweyh, H.-J., Weber, N., and Schuster, S.C. (2011) Integrative analysis of environmental sequences using MEGAN4. *Genome Res* **21**: 1552–1560.

Oono, R., Rasmussen, A., and Lefèvre, E. (2017) Distance decay relationships in foliar fungal endophytes are driven by rare taxa. *Environ Microbiol* **19**: 2794–2805.

Toju, H., Hirokazu, T., Tanabe, A.S., Satoshi, Y., and Hirotoshi, S. (2012) High-coverage ITS primers for the DNA-based identification of Ascomycetes and Basidiomycetes in environmental samples. *PLoS One* **7**: e40863.
