# Supplementary material for: How do phytophagous insects affect phyllosphere fungi? Tracking fungi from milkweed to monarch caterpillar frass reveals communities dominated by fungal yeast
Source: Environ Microbiol Rep. 2024 May 13;16(3):e13213. doi: 10.1111/1758-2229.13213 (PMC11089944; doi:10.1111/1758-2229.13213)
Supplement: Supplementary file 4 — APPENDIX S4. R script for all community analyses. [file EMI4-16-e13213-s005.pdf]

Monarch

Ryoko Oono & Mari Irving, UCSB

1/20/2021

```
chooseCRANmirror(graphics=FALSE, ind=1)
knitr::opts_chunk$set(echo = TRUE, warning = FALSE,tidy.opts=list(width.cutoff=60),tidy=TRUE)

#install.packages("phyloseq")

library(stringr)
library(phyloseq)
library(metagenomeSeq)
```

```
## Loading required package: Biobase

## Loading required package: BiocGenerics

## Loading required package: parallel

##
## Attaching package: 'BiocGenerics'

## The following objects are masked from 'package:parallel':
##
##     clusterApply, clusterApplyLB, clusterCall, clusterEvalQ,
##     clusterExport, clusterMap, parApply, parCapply, parLapply,
##     parLapplyLB, parRapply, parSapply, parSapplyLB

## The following objects are masked from 'package:stats':
##
##     IQR, mad, sd, var, xtabs

## The following objects are masked from 'package:base':
##
##     anyDuplicated, append, as.data.frame, basename, cbind, colnames,
##     dirname, do.call, duplicated, eval, evalq, Filter, Find, get, grep,
##     grepl, intersect, is.unsorted, lapply, Map, mapply, match, mget,
##     order, paste, pmax, pmax.int, pmin, pmin.int, Position, rank,
##     rbind, Reduce, rownames, sapply, setdiff, sort, table, tapply,
##     union, unique, unsplit, which, which.max, which.min

## Welcome to Bioconductor
##
##     Vignettes contain introductory material; view with
##     'browseVignettes()'. To cite Bioconductor, see
##     'citation("Biobase")', and for packages 'citation("pkgname")'.

##
## Attaching package: 'Biobase'

## The following object is masked from 'package:phyloseq':
```

```
##
##     sampleNames
## Loading required package: limma
##
## Attaching package: 'limma'
## The following object is masked from 'package:BiocGenerics':
##
##     plotMA
## Loading required package: glmnet
## Loading required package: Matrix
## Loaded glmnet 4.0-2
## Loading required package: RColorBrewer
```

```r
library(ggplot2)
library(vegan)
```

```
## Loading required package: permute
## Loading required package: lattice
## This is vegan 2.5-6
```

```r
#library(plyr)
#remotes::install_github("adrientaudiere/MiscMetabar")
library(MiscMetabar)
```

```
## Loading required package: grid
## Loading required package: dada2
## Loading required package: Rcpp
## Loading required package: magrittr
##
## Attaching package: 'MiscMetabar'
## The following object is masked from 'package:phyloseq':
##
##     phyloseq_to_deseq2
```

```r
library(venneuler)
```

```
## Loading required package: rJava
```

```r
library(eulerr)
```

```
## Registered S3 method overwritten by 'eulerr':
##   method    from
##   plot.venn gplots
```

```r
#install.packages("indicspecies")
library(indicspecies)
#install.packages("here")
library(here)
```

```
## here() starts at /Users/ryokooono/Dropbox/R_projects/Monarch/Monarch
```

```
library(ggpubr)
source(here::here("Script", "Functions", "make_metagenomeSeq.R"))
source(here::here("Script", "Functions","ps_venn.R"))
source(here::here("Script", "Functions","bv.step.R"))
source(here::here("Script", "Functions","bio.env.R"))

library(dplyr)
```

```
##
## Attaching package: 'dplyr'

## The following object is masked from 'package:Biobase':
##
##     combine

## The following objects are masked from 'package:BiocGenerics':
##
##     combine, intersect, setdiff, union

## The following objects are masked from 'package:stats':
##
##     filter, lag

## The following objects are masked from 'package:base':
##
##     intersect, setdiff, setequal, union
```

```
library(scales)
library(reshape2)
library(purrr)
```

```
##
## Attaching package: 'purrr'

## The following object is masked from 'package:scales':
##
##     discard

## The following object is masked from 'package:magrittr':
##
##     set_names
```

We will upload the three objects that make up a phyloseq object, the OTU or ASV table, the metadata, and the taxonomic assignment file. The metadata file includes plant ID, caterpillar ID, sampling dates, library ID, and preceding or following frass sample IDs.

```
OTU_table <- as.matrix(read.csv(here::here("Data", "monarch_fungiOTU.csv"),
    header = TRUE, row.names = 1, sep = ","))  #Upload the OTU table
OTU_tab <- otu_table(OTU_table, taxa_are_rows = TRUE)
sample_table <- read.csv(here::here("Data", "Monarch_metadata_AppendixS3.csv"),
    header = TRUE, row.names = 1, sep = ",")  #Upload the metada table
sample_tab <- sample_data(sample_table)
taxa_table <- as.matrix(read.csv(here::here("Data", "Monarchtaxa_v2.csv"),
    header = TRUE, row.names = 1, sep = ","))  #Upload the taxa data if you have this
taxa_tab <- tax_table(taxa_table)
monarch_phylo <- phyloseq(OTU_tab, sample_tab, taxa_tab)  #construct the components of a phyloseq objec
```

We have now made the phyloseq object monarch_phylo. These samples were run across two sequencing libraries, which could introduce non-biological variations into the data (different sequencing libraries might

produce difference sequences). Hence, will first test to see if the two libraries (which contained some of the same samples) are significantly different from one another (i.e., spatially separated on an nMDS and test with permanova). We must first take out any samples with low read counts.

```
# Have to delete samples with only 0 or 1 taxon.
richness_monarch <- estimate_richness(monarch_phylo, measures = "Observed")
# there are samples with only 1 or 0 OTUs.
monarch_otus <- richness_monarch[which(richness_monarch$Observed >=
    2), , drop = FALSE]
monarch_phylo_01 <- prune_samples(rownames(monarch_otus), monarch_phylo)

# make nMDS (non-metric Multidimensional Scaling)
Monarch_meta <- make_metagenomeSeq(monarch_phylo_01)
```

```
## Default value being used.
```

```
Monarch_CSSmat = MRcounts(Monarch_meta, norm = TRUE, log = TRUE)
Monarch_tCSSmat <- t(Monarch_CSSmat)
Monarch_BCData <- vegdist(Monarch_tCSSmat, method = "bray", binary = FALSE,
    diag = TRUE, upper = TRUE)

set.seed(53)
Monarch_nMDS <- metaMDS(Monarch_BCData, distance = "bray", k = 2,
    trymax = 1000)

# Using the scores function from vegan to extract the site
# scores and convert to a data.frame

Monarch.data.scores <- as.data.frame(scores(Monarch_nMDS))

Monarch.data.scores$library <- Monarch_meta$Library  #create a column of site names, from the rownames
```

We will make the nMDS plot but we are performing a PERMANOVA to see if samples from the two libraries are significantly different from each other.

```
Monarch_nmds_plot<-ggplot(data=Monarch.data.scores, aes(NMDS1,NMDS2, shape = library)) +
  geom_point(aes(x=NMDS1,y=NMDS2,fill = library),size=4, color = "black") + # add the point markers
    scale_shape_manual(values=c(21, 23), name = "Library", guide = FALSE) +
scale_fill_manual(values=c("brown","darkgreen" ), name = "", guide = FALSE) +
 guides(name = "Library",fill = guide_legend(override.aes = list(shape =c(21, 23)))) +
       # shape = guide_legend(override.aes = list(fill = c("brown","darkgreen")))) +
 # coord_equal(xlim = c(-0.6,0.6), ylim =c(-0.6,0.55)) +
  theme(axis.title = element_text(face = "plain",size = 15)) +
  theme(axis.text = element_text(size = 10)) +
  theme(axis.title.x=element_text(margin=margin(10,10,10,10))) +
  theme_bw() +
  theme(panel.grid.major = element_blank(),panel.grid.minor = element_blank()) +
  labs(x = "nMDS 1",y = "nMDS 2") +
  theme(legend.text=element_text(size=12), legend.title = element_text(size=15),
        legend.position = "right",aspect.ratio=1) +
  stat_ellipse(type = "t", level = 0.68)

Monarch_BC <- adonis(Monarch_BCData~ Monarch_meta$Library, permutations = 1000, method="bray")
Monarch_nmds_plot
```

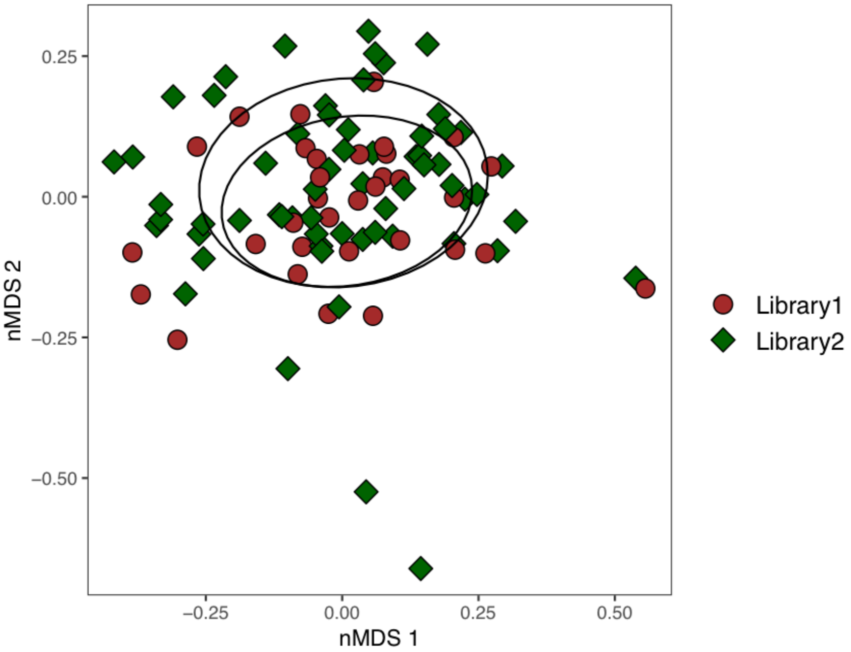


```
Monarch_BC
```

```
##
## Call:
## adonis(formula = Monarch_BCData ~ Monarch_meta$Library, permutations = 1000,      method = "bray")
##
## Permutation: free
## Number of permutations: 1000
##
## Terms added sequentially (first to last)
##
##                      Df SumsOfSqs MeanSqs F.Model      R2 Pr(>F)
## Monarch_meta$Library  1    0.2255 0.22554 0.71947 0.00768 0.8681
## Residuals            93   29.1531 0.31347         0.99232
## Total                94   29.3786                 1.00000
```

```
png(here::here("Output", "Monarch_nmds_library.png"), units="in", width=5, height=5, res=300)


Monarch_nmds_plot
dev.off()
```

```
## pdf
##   2
```

```
#Yay, not different between libraries!!!
```

Now that we know that the libraries did not produce significantly different data, we will use merge_samples function to merge the reads from all of the same samples from different and same libraries.

```
dup_monarch <- duplicated(sample_data(monarch_phylo)$Sample)
unique_samples <- sample_data(monarch_phylo)[!dup_monarch]
dup_sample <- prune_samples(sample_names(unique_samples), monarch_phylo)
merged_replicates <- merge_samples(monarch_phylo, "Sample")
merged_sample <- as.data.frame(unclass(sample_data(dup_sample)))
row.names(merged_sample) <- sample_names(merged_replicates)

phyloseq_02 <- phyloseq(otu_table(merged_replicates), sample_data(merged_sample),
    tax_table(merged_replicates))  #this creates new phyloseq object where same samples from the two li

phyloseq_03 <- subset_samples(phyloseq_02, material_species !=
    "na")  #take out clone

sample_sums(phyloseq_03)  #see where you want to cut off for low reads
```

```
##    A2_3   A5_2 A5_2_1   A7_4   A7_5   B2_2   B4_2   B5_2 B5_2_1   B7_4   B7_5
##      52     45  24975  85710     33   6609 230460 111345   1728  40650  56293
##    C2_2   C3_2   C5_2   C7_3   C7_5   D2_2   D3_2   D4_3   D5_2 D5_2_1   D7_3
##   34610  24991  58730  53569 102594   9461     67  94738  77276 193855  59781
##    D7_4   D7_5   E2_2   E2_3   E5_2   E7_5   F2_2   F5_2   F7_5   G2_2   G3_2
## 198079  78946  22657    188 109800 127313  31428  30165   2154  16985  22523
## G5_2_1   G7_3   H2_2   H3_3   H4_2   H5_2 H5_2_1   H7_3   H7_4   H7_5   I2_2
##   12246  35215  12155  49740     52 269541  55493  73376    127 159877  36880
##    I4_2   I5_1 I5_2_1   I7_4   I7_5   J5_1   J7_5   K3_2   K5_1   K7_5
##   33167  22490 344445  79019  25801     16  54176 144556  20708 144716
```

```
hist(sample_sums(phyloseq_03), breaks = 10000, xlim = c(0, 500))
```

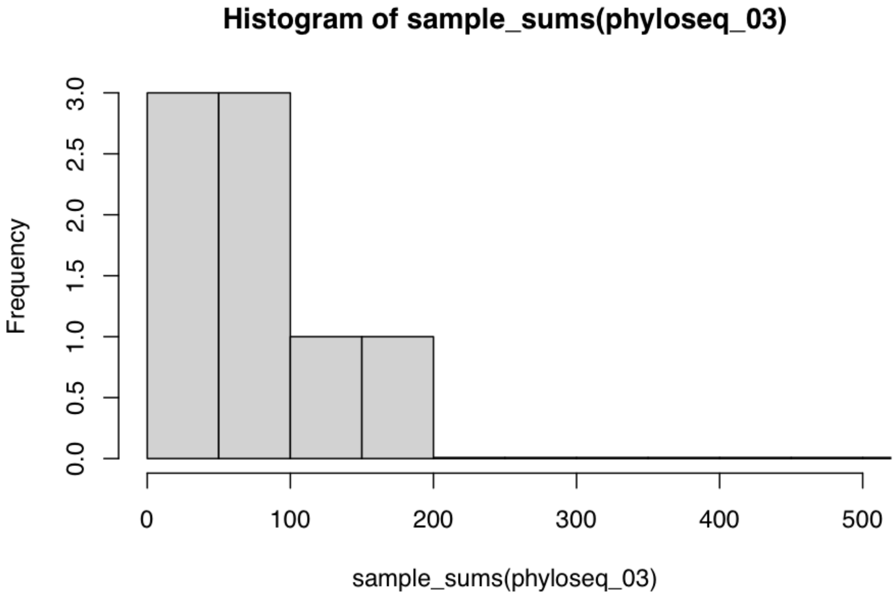


```
phyloseq_04 = prune_samples(sample_sums(phyloseq_03) >= 300,
    phyloseq_03)  #prune samples with less than 300 reads, this has 2032 taxa
```

```r
phylo_rarefy <- rarefy_even_depth(phyloseq_04, verbose = TRUE,
    rngseed = 526)
```

```
## `set.seed(526)` was used to initialize repeatable random subsampling.

## Please record this for your records so others can reproduce.

## Try `set.seed(526); .Random.seed` for the full vector

## ...

## 788OTUs were removed because they are no longer
## present in any sample after random subsampling

## ...
```

```r
# this has 1230 taxa
```

We have taken out the positive control (clones) and now we are checking to see which samples have low read numbers that may want to eliminate from final analyses. Based on the histogram, we assessed that a cut-off of 300 reads per sample separated most samples into high and low read numbers. Pruning samples at this cut-off resulted in the loss of 8 samples but we retained all taxa (2032 ASVs). We then rarefied these down to a common sequencing depth of 1728 reads. This led to the loss of 802 ASVs.

We will now manipulate the data (subset) in order to obtain a descriptive summary of each group. For one, we must merge samples from the same caterpillars. Once they were merged, we also rarefied these samples once again because frass from some caterpillars were sampled more times than others. We then tested to see if the frass from caterpillars eating one milkweed species were different from the other milkweed species.

```r
phylo_frass <- subset_samples(phylo_rarefy, Material == "Frass")
phylo_frass_per_caterpillar <- merge_samples(phylo_frass, "Caterpillar")

phylo_frass_per_caterpillar_rare <- rarefy_even_depth(phylo_frass_per_caterpillar,
    verbose = TRUE, rngseed = 526)
```

```
## `set.seed(526)` was used to initialize repeatable random subsampling.

## Please record this for your records so others can reproduce.

## Try `set.seed(526); .Random.seed` for the full vector

## ...

## 786OTUs were removed because they are no longer
## present in any sample after random subsampling

## ...
```

```r
sample_data(phylo_frass_per_caterpillar_rare)$Milkweed_species <- c("fascicularis",
    "fascicularis", "fascicularis", "curassavica", "curassavica",
    "fascicularis", "fascicularis", "curassavica", "curassavica",
    "curassavica")
# merging caterpillar samples loses the metadata file so the
# host sample data was manually added later
Frass_meta <- make_metagenomeSeq(phylo_frass_per_caterpillar_rare)
```

```
## Default value being used.
```

```r
Frass_CSSmat = MRcounts(Frass_meta, norm = TRUE, log = TRUE)
Frass_tCSSmat <- t(Frass_CSSmat)
Frass_BCData <- vegdist(Frass_tCSSmat, method = "bray", binary = FALSE,
    diag = TRUE, upper = TRUE)
```

```r
set.seed(53)

Frass_permanova <- adonis(Frass_BCData ~ Frass_meta$Milkweed_species,
    permutations = 1000, method = "bray")
```

We find that they are statistically significant R^2 = 0.138, p = 0.0289.

This next chunk divides up the data into subsets by the four treatment groups and also prepares the phyloseq object to be used in the indicator species analysis. We cannot use phyloseq_04 because that object contains multiple samples from the same caterpillar.

```r
phylo_fasc_leaf <- subset_samples(phylo_rarefy, material_species ==
    "plant_fascicularis")
phylo_curr_leaf <- subset_samples(phylo_rarefy, material_species ==
    "plant_currasavica")

phylo_fasc_frass <- subset_samples(phylo_rarefy, material_species ==
    "frass_fascicularis")
phylo_fasc_frass_per_caterpillar <- merge_samples(phylo_fasc_frass,
    "Caterpillar")
phylo_fasc_frass_per_caterpillar_rare <- rarefy_even_depth(phylo_fasc_frass_per_caterpillar,
    verbose = TRUE, rngseed = 526)
```

```
## `set.seed(526)` was used to initialize repeatable random subsampling.

## Please record this for your records so others can reproduce.

## Try `set.seed(526); .Random.seed` for the full vector

## ...

## 1067OTUs were removed because they are no longer
## present in any sample after random subsampling

## ...
```

```r
phylo_curr_frass <- subset_samples(phylo_rarefy, material_species ==
    "frass_currasavica")
phylo_curr_frass_per_caterpillar <- merge_samples(phylo_curr_frass,
    "Caterpillar")
phylo_curr_frass_per_caterpillar_rare <- rarefy_even_depth(phylo_curr_frass_per_caterpillar,
    verbose = TRUE, rngseed = 526)
```

```
## `set.seed(526)` was used to initialize repeatable random subsampling.

## Please record this for your records so others can reproduce.

## Try `set.seed(526); .Random.seed` for the full vector

## ...

## 817OTUs were removed because they are no longer
## present in any sample after random subsampling

## ...
```

```r
sample_data(phylo_fasc_frass_per_caterpillar)$material_species <- "frass_fascicularis"
sample_data(phylo_curr_frass_per_caterpillar)$material_species <- "frass_curassavica"

indicator_rare_phylo <- merge_phyloseq(phylo_fasc_leaf, phylo_curr_leaf,
    phylo_fasc_frass_per_caterpillar, phylo_curr_frass_per_caterpillar)
```

indicator_rare_phylo is made, which has only 27 samples. There is 5 caterpillar frass samples that ate A. curassavica, 5 that ate A. fascicularis, 9 A. curassavica leaves, and 8 A fascicularis leaves.

This next chunk produces .csv files of the ASV files specific to each treatment group.

```
write.csv(t(otu_table(phylo_fasc_leaf)), file = here::here("Data",
    "fasc_leaf_merged_ASV_table.csv"))
write.csv(t(otu_table(phylo_curr_leaf)), file = here::here("Data",
    "curr_leaf_merged_ASV_table.csv"))
write.csv(t(otu_table(phylo_fasc_frass_per_caterpillar_rare)),
    file = here::here("Data", "fasc_frass_merged_ASV_table.csv"))
write.csv(t(otu_table(phylo_curr_frass_per_caterpillar_rare)),
    file = here::here("Data", "curr_frass_merged_ASV_table.csv"))
```

We prune taxa that have at least 100 reads and make nMDS with this new phyloseq object, phyloseq_04_100

```
phyloseq_04_100 <- prune_taxa(taxa_sums(phyloseq_04) > 100, phyloseq_04)

Monarch_merged_meta <- sample_data(phyloseq_04_100)

Monarch_merged_meta <- make_metagenomeSeq(phyloseq_04_100)
```

```
## Default value being used.
```

```
Monarch_merged_CSSmat = MRcounts(Monarch_merged_meta, norm = TRUE,
    log = TRUE)
Monarch_merged_tCSSmat <- t(Monarch_merged_CSSmat)
Monarch_merged_BCData <- vegdist(Monarch_merged_tCSSmat, method = "bray",
    binary = FALSE, diag = TRUE, upper = TRUE)

set.seed(53)
Monarch_merged_nMDS <- metaMDS(Monarch_merged_BCData, distance = "bray",
    k = 2, trymax = 1000)
```

```
## Run 0 stress 0.1813578
## Run 1 stress 0.1918879
## Run 2 stress 0.19362
## Run 3 stress 0.1850053
## Run 4 stress 0.181332
## ... New best solution
## ... Procrustes: rmse 0.002902182  max resid 0.01458792
## Run 5 stress 0.2261212
## Run 6 stress 0.2077377
## Run 7 stress 0.1850587
## Run 8 stress 0.1813574
## ... Procrustes: rmse 0.002847289  max resid 0.0141362
## Run 9 stress 0.1980357
## Run 10 stress 0.2173887
## Run 11 stress 0.221007
## Run 12 stress 0.1918574
## Run 13 stress 0.2071384
## Run 14 stress 0.2156393
## Run 15 stress 0.1850311
## Run 16 stress 0.2186102
## Run 17 stress 0.2049312
```

```
## Run 18 stress 0.2138308
## Run 19 stress 0.1907681
## Run 20 stress 0.2310544
## Run 21 stress 0.2428961
## Run 22 stress 0.2093644
## Run 23 stress 0.2031977
## Run 24 stress 0.2049304
## Run 25 stress 0.2173015
## Run 26 stress 0.2109386
## Run 27 stress 0.1850042
## Run 28 stress 0.1964234
## Run 29 stress 0.1907523
## Run 30 stress 0.2066927
## Run 31 stress 0.4017152
## Run 32 stress 0.1982725
## Run 33 stress 0.2322438
## Run 34 stress 0.1963623
## Run 35 stress 0.2010913
## Run 36 stress 0.2190566
## Run 37 stress 0.1813573
## ... Procrustes: rmse 0.002807032  max resid 0.01402729
## Run 38 stress 0.2067134
## Run 39 stress 0.1813576
## ... Procrustes: rmse 0.002934902  max resid 0.01469573
## Run 40 stress 0.205823
## Run 41 stress 0.2086578
## Run 42 stress 0.2236141
## Run 43 stress 0.2086579
## Run 44 stress 0.2282007
## Run 45 stress 0.1850301
## Run 46 stress 0.2401715
## Run 47 stress 0.2189964
## Run 48 stress 0.2242255
## Run 49 stress 0.1963627
## Run 50 stress 0.4017134
## Run 51 stress 0.2037766
## Run 52 stress 0.2113216
## Run 53 stress 0.2000382
## Run 54 stress 0.1970479
## Run 55 stress 0.2079467
## Run 56 stress 0.2164253
## Run 57 stress 0.2049304
## Run 58 stress 0.1919206
## Run 59 stress 0.236633
## Run 60 stress 0.2171204
## Run 61 stress 0.1904953
## Run 62 stress 0.2191421
## Run 63 stress 0.1813579
## ... Procrustes: rmse 0.002874122  max resid 0.01442633
## Run 64 stress 0.2368627
## Run 65 stress 0.1904942
## Run 66 stress 0.2043513
## Run 67 stress 0.2086539
## Run 68 stress 0.1918575
```

```
## Run 69 stress 0.2171211
## Run 70 stress 0.1981267
## Run 71 stress 0.2013694
## Run 72 stress 0.2000164
## Run 73 stress 0.2183797
## Run 74 stress 0.1918576
## Run 75 stress 0.2091465
## Run 76 stress 0.1907522
## Run 77 stress 0.1918186
## Run 78 stress 0.2304334
## Run 79 stress 0.2118939
## Run 80 stress 0.191819
## Run 81 stress 0.2086587
## Run 82 stress 0.1852325
## Run 83 stress 0.2134532
## Run 84 stress 0.2275903
## Run 85 stress 0.212319
## Run 86 stress 0.2172285
## Run 87 stress 0.2165236
## Run 88 stress 0.1918575
## Run 89 stress 0.2071388
## Run 90 stress 0.2377397
## Run 91 stress 0.1935972
## Run 92 stress 0.1813574
## ... Procrustes: rmse 0.002838476  max resid 0.0141962
## Run 93 stress 0.2110615
## Run 94 stress 0.1813316
## ... New best solution
## ... Procrustes: rmse 0.0001329808  max resid 0.0005882409
## ... Similar to previous best
## *** Solution reached
```

```
Monarch_merged.data.scores <- as.data.frame(scores(Monarch_merged_nMDS))

Monarch_merged.data.scores$material <- Monarch_merged_meta$Material
Monarch_merged.data.scores$caterpillar <- Monarch_merged_meta$Caterpillar
Monarch_merged.data.scores$date <- Monarch_merged_meta$Date_collected
Monarch_merged.data.scores$milkweed <- Monarch_merged_meta$Milkweed_species
Monarch_merged.data.scores$next1 <- Monarch_merged_meta$PoopAfter1
Monarch_merged.data.scores$next2 <- Monarch_merged_meta$PoopAfter2
Monarch_merged.data.scores$next3 <- Monarch_merged_meta$PoopAfter3
Monarch_merged.data.scores$next4 <- Monarch_merged_meta$PoopAfter4

Monarch_merged.data.scores$LeafPoopAfter <- Monarch_merged_meta$LeafPoopAfter

Monarch_merged.data.scores$milkweed_material <- paste(Monarch_merged.data.scores$material,
    "_", Monarch_merged.data.scores$milkweed)




Just_plant <- subset_samples(phyloseq_04_100, Material == "Plant")

Just_plant_meta <- make_metagenomeSeq(Just_plant)
```

```
## Default value being used.
```

```r
Just_plant_meta_CSSmat = MRcounts(Just_plant_meta, norm = TRUE,
    log = TRUE)
Just_plant_meta_CSSmat_tCSSmat <- t(Just_plant_meta_CSSmat)
Just_plant_BCData <- vegdist(Just_plant_meta_CSSmat_tCSSmat,
    method = "bray", binary = FALSE, diag = TRUE, upper = TRUE)

Just_plant_BC_material <- adonis(Just_plant_BCData ~ Just_plant_meta$Milkweed_species,
    permutations = 1000, method = "bray")

betadisper(Monarch_merged_BCData, Monarch_merged.data.scores$milkweed_material)
```

```
##
##  Homogeneity of multivariate dispersions
##
## Call: betadisper(d = Monarch_merged_BCData, group =
## Monarch_merged.data.scores$milkweed_material)
##
## No. of Positive Eigenvalues: 44
## No. of Negative Eigenvalues: 1
##
## Average distance to median:
##  Frass _ A_currasavica Frass _ A_fascicularis  Plant _ A_currasavica
##                 0.4984                 0.4859                 0.3771
## Plant _ A_fascicularis
##                 0.3584
##
## Eigenvalues for PCoA axes:
## (Showing 8 of 45 eigenvalues)
##  PCoA1  PCoA2  PCoA3  PCoA4  PCoA5  PCoA6  PCoA7  PCoA8
## 1.7923 1.3384 0.9165 0.6882 0.5695 0.5054 0.4395 0.4243
```

```r
Monarch_BC_material <- adonis(Monarch_merged_BCData ~ Monarch_merged_meta$Material *
    Monarch_merged_meta$Milkweed_species, permutations = 1000,
    method = "bray")
Monarch_nmds_plot
```

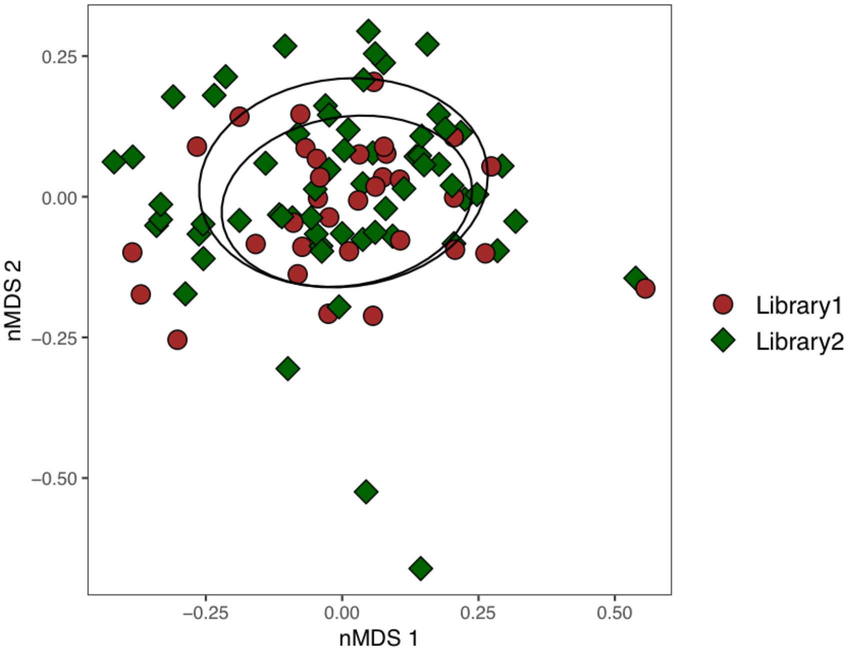


```
Monarch_BC
```

```
##
## Call:
## adonis(formula = Monarch_BCData ~ Monarch_meta$Library, permutations = 1000,      method = "bray")
##
## Permutation: free
## Number of permutations: 1000
##
## Terms added sequentially (first to last)
##
##                      Df SumsOfSqs MeanSqs F.Model      R2 Pr(>F)
## Monarch_meta$Library  1    0.2255 0.22554 0.71947 0.00768 0.8681
## Residuals            93   29.1531 0.31347         0.99232
## Total                94   29.3786                 1.00000
```

```
phylo_rarefied_pair_plant <- subset_samples(phylo_rarefy, LeafPoopAfter !=
    "")

sample_data(phylo_rarefy)$LeafPoopAfter
```

```
##  [1] ""      "A2_3" ""      ""      ""      ""      "B4_2" "B5_2" ""      ""
## [11] ""      "C3_2" "C5_2" ""      ""      ""      ""      "D3_2" "D4_2" "D5_2"
## [21] ""      ""      "E5_2" ""      ""      "F5_2" ""      ""      ""      "G3_2"
## [31] ""      ""      ""      ""      "H3_3" "H5_2" ""      ""      ""      ""
## [41] "I4_2" "I5_1" "J5_1" ""      ""      "K5_1"
```

```
phylo_rarefied_pair_frass <- prune_samples(sample_data(phylo_rarefy)$LeafPoopAfter,
    phylo_rarefy)

pair_plant <- estimate_richness(phylo_rarefied_pair_plant)
pair_frass <- estimate_richness(phylo_rarefied_pair_frass)



rownames(pair_plant) <- sample_data(phylo_rarefied_pair_plant)$LeafPoopAfter
pairs <- merge(pair_plant, pair_frass, by = 0, all = TRUE)

paired_richness_graph <- ggpaired(pairs, cond1 = "Observed.x",
    cond2 = "Observed.y", fill = "condition", alpha = 0.5) +
    labs(y = "Species richness", x = "") + theme(axis.text.x = element_blank()) +
    scale_fill_manual(values = c("chartreuse4", "chocolate4")) +
    guides(fill = FALSE)

png(here::here("Output", "Pairedrichness.png"), units = "in",
    width = 4, height = 4, res = 300)
paired_richness_graph
dev.off()
```

```
## pdf
##   2
```

```
paired_shannon_graph <- ggpaired(pairs, cond1 = "Shannon.x",
    cond2 = "Shannon.y", fill = "condition", alpha = 0.5) + labs(y = "Shannon's diversity",
    x = "") + theme(axis.text.x = element_blank()) + scale_fill_manual(values = c("chartreuse4",
    "chocolate4")) + guides(fill = FALSE)


Plant_Rarefy <- subset_samples(phylo_rarefy, Material == "Plant")
Frass_Rarefy <- subset_samples(phylo_rarefy, Material == "Frass")
Frass_caterpillar <- merge_samples(Frass_Rarefy, "Caterpillar")

Frass_caterpillar_rarefied <- rarefy_even_depth(Frass_caterpillar,
    verbose = TRUE, rngseed = 526)
```

```
## `set.seed(526)` was used to initialize repeatable random subsampling.

## Please record this for your records so others can reproduce.

## Try `set.seed(526); .Random.seed` for the full vector

## ...

## 786OTUs were removed because they are no longer
## present in any sample after random subsampling

## ...
```

```
Frass_rich <- estimate_richness(Frass_caterpillar_rarefied)
Plant_rich <- estimate_richness(Plant_Rarefy)

row.names.remove <- c("Hanan")

Frass_rich_no_outlier <- Frass_rich[!(row.names(Frass_rich) %in%
```

```
    row.names.remove), ]
t.test(Frass_rich_no_outlier$Observed, Plant_rich$Observed)
```

```
##
##  Welch Two Sample t-test
##
## data:  Frass_rich_no_outlier$Observed and Plant_rich$Observed
## t = -2.7581, df = 22.844, p-value = 0.01124
## alternative hypothesis: true difference in means is not equal to 0
## 95 percent confidence interval:
##  -54.076614  -7.714236
## sample estimates:
## mean of x mean of y
##  60.22222  91.11765
```

```
Venn <- ps_venn(phyloseq_04_100, "material_species")
Venn_counts <- cbind(rownames(Venn$data$centers),Venn$data$centers$quantities)
row.names(Venn_counts) <- Venn_counts[,1]
Venn_label<-Venn_counts[which(row.names(Venn_counts) == c("frass_currasavica",
"frass_fascicularis",
"plant_currasavica",
"plant_fascicularis",
"frass_currasavica&frass_fascicularis&plant_currasavica&plant_fascicularis")),]

Venn_plot<- venn_phyloseq(phyloseq_04_100, "material_species",print_values =  FALSE) +
    scale_fill_manual(values=c("#996633","#CC9933","#006633","#8DA03C"), name = "",
                    labels = c(expression(paste("Frass ", italic("A. curassavica"))),



  theme(legend.text.align = 0) +
    geom_label(aes(label=Venn_label[1,2],
    x=0.57, y=0.27), #A. curassavica frass
    label.padding = unit(0.5, "lines"), # Rectangle size around label
    label.size = 0.2,size = 6,
    color = "black",fill="#996633", alpha = 0.005) +

    geom_label(aes(label=Venn_label[2,2],
    x=0.285, y=0.485), #A. fascicularis frass
    label.padding = unit(0.5, "lines"), # Rectangle size around label
    label.size = 0.2,size = 6,
    color = "black",fill="#996633", alpha = 0.005) +

    geom_label(aes(label=Venn_label[3,2],
    x=0.67, y=0.67), #A. curassavica plant
    label.padding = unit(0.5, "lines"), # Rectangle size around label
    label.size = 0.2,size = 6,
    color = "black",fill="#996633", alpha = 0.005) +

    geom_label(aes(label=Venn_label[4,2],
    x=0.405, y=0.665), #A. fascicularis plant
    label.padding = unit(0.3, "lines"), # Rectangle size around label
    label.size = 0.13,size = 3,
    color = "black",fill="#996633", alpha = 0.005) +
```

```
    geom_label(aes(label=Venn_label[5,2],
    x=0.46, y=0.51), #everything
    label.padding = unit(0.5, "lines"), # Rectangle size around label
    label.size = 0.2,size = 6,
    color = "black",fill="#996633", alpha = 0.005) +

    geom_label(aes(label=Venn_counts[6,2],
     x=0.69, y=0.5), #just curassavica
    label.padding = unit(0.5, "lines"), # Rectangle size around label
    label.size = 0.2,size = 6,
    color = "black",fill="#996633", alpha = 0.005) +

    geom_label(aes(label=Venn_counts[9,2],
     x=0.36, y=0.57), #just fascicularis
    label.padding = unit(0.3, "lines"), # Rectangle size around label
    label.size = 0.1,size = 4,
    color = "black",fill="#996633", alpha = 0.005) +

    geom_label(aes(label=Venn_counts[10,2],
     x=0.5, y=0.65), #just plants
    label.padding = unit(0.5, "lines"), # Rectangle size around label
    label.size = 0.1,size = 6,
    color = "black",fill="#996633", alpha = 0.005) +

    geom_label(aes(label=Venn_counts[5,2],
     x=0.365, y=0.38), #just frass
    label.padding = unit(0.3, "lines"), # Rectangle size around label
    label.size = 0.09, size = 3,
    color = "black",fill="#996633", alpha = 0.005)



Venn_plot
```

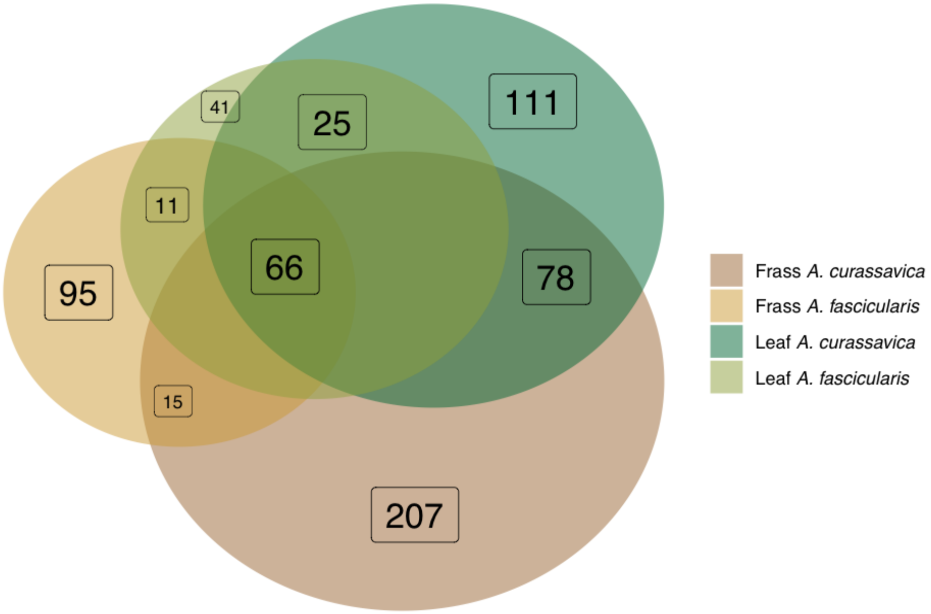


```
b <- ggplot_build(Venn_plot)
b$layout$panel_params[[1]]$x.range
```

```
## [1] 0.1929113 0.8093628
```

```
b$layout$panel_params[[1]]$y.range
```

```
## [1] 0.1477535 0.7937373
```

```
#this tells you the x-y coordinates to put the labels

png(here::here("Output", "Monarch_venn.png"), units="in", width=7, height=5, res=300)

Venn_plot

dev.off()
```

```
## pdf
##   2
```

```
Monarch_X0 <- subset(Monarch_merged.data.scores, Monarch_merged.data.scores$LeafPoopAfter != "")

nmds1_x1 <- list()
nmds2_x1 <- list()

for (i in 1:dim(Monarch_X0)[1]){
  if (Monarch_X0$LeafPoopAfter[i] %in% rownames(Monarch_merged.data.scores)){
  nmds1_x1[i]<- Monarch_merged.data.scores[which(rownames(Monarch_merged.data.scores) == Monarch_X0$Lea
  nmds2_x1[i]<- Monarch_merged.data.scores[which(rownames(Monarch_merged.data.scores) == Monarch_X0$Lea
  } else {
  nmds1_x1[i] <- NA
```

```
  nmds2_x1[i] <- NA
  }
}

Monarch_X0$x1_nmds1<-unlist(nmds1_x1)
Monarch_X0$x1_nmds2<-unlist(nmds2_x1)


Tracking_nmds_plot<-ggplot(data=Monarch_merged.data.scores, aes(NMDS1,NMDS2,fill = milkweed_material)) ·
  geom_point(aes(x=NMDS1,y=NMDS2,fill = milkweed_material, shape = milkweed), size=4, color = "black") ·
scale_shape_manual(values=c(23,21), name = "Milkweed", guide = FALSE) +
  scale_fill_manual(values=c("#996633","#CC9933","#006633","#8DA03C"), name = "", guide = FALSE,
                  labels = c(expression(paste("Frass ", italic("A. curassavica"))),



  guides(fill = guide_legend(override.aes = list(shape = c(23,21)))) +
 # geom_line(arrow = arrow()) + # spiders
  geom_segment(data = Monarch_X0, aes(x = NMDS1, y = NMDS2, xend = x1_nmds1, yend = x1_nmds2), alpha = (
    theme_bw() +
 # coord_equal(xlim = c(-0.6,0.6), ylim =c(-0.6,0.55)) +
  theme(axis.title = element_text(face = "plain",
                                size = 15,
                                margin=margin(10,10,10,10)),
        axis.text = element_text(size = 10),
        panel.grid.major = element_blank(),
        panel.grid.minor = element_blank(),
        legend.text=element_text(size=12),
        legend.text.align = 0,
        legend.title = element_text(size=15),
        legend.position = "right",aspect.ratio=1) +
  labs(x = "nMDS 1", y = "nMDS 2")

Tracking_nmds_plot
```

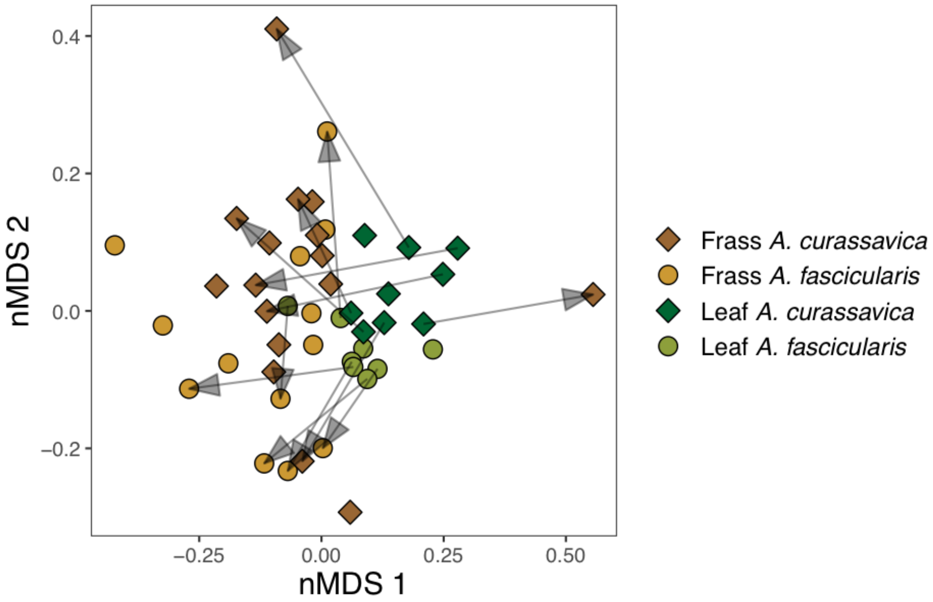


```
png(here::here("Output", "Monarch_nmds_track_v2.png"), units="in", width=7, height=7, res=300)


Tracking_nmds_plot

dev.off()
```

```
## pdf
##   2
```

```
Final_nmds_plot<-ggplot(data=Monarch_merged.data.scores, aes(NMDS1,NMDS2, fill = milkweed_material)) +
  geom_point(aes(x=NMDS1,y=NMDS2,fill = milkweed_material, shape = milkweed), size=4, color = "black") +
scale_shape_manual(values=c(23,21), name = "Milkweed", guide = FALSE) +
  scale_fill_manual(values=c("#996633","#CC9933","#006633","#8DA03C"), name = "", guide = FALSE,
                  labels = c(expression(paste("Frass ", italic("A. curassavica"))),



  guides(fill = guide_legend(override.aes = list(shape = c(23,21)))) +
 # geom_line(arrow = arrow()) + # spiders
    theme_bw() +
 # coord_equal(xlim = c(-0.6,0.6), ylim =c(-0.6,0.55)) +
  theme(axis.title = element_text(face = "plain",
                                  size = 15,
                                  margin=margin(10,10,10,10)),
        axis.text = element_text(size = 10),
        panel.grid.major = element_blank(),
        panel.grid.minor = element_blank(),
        legend.text=element_text(size=12),
        legend.text.align = 0,
```

```
        legend.title = element_text(size=15),
        legend.position = "right",aspect.ratio=1) +
  labs(x = "nMDS 1", y = "nMDS 2") +
    stat_ellipse(type = "t", level = 0.68)

png(here::here("Output", "Monarch_nmds_final.png"), units="in", width=7, height=7, res=300)

Final_nmds_plot

dev.off()
```

```
## pdf
##   2
```

```
monarch_relative <- transform_sample_counts(phylo_rarefy, function(x) x/sum(x))
monarch_rarefy_count <- transform_sample_counts(phylo_rarefy,
    function(x) {
        x[x > 0] <- 1
        return(x)
    })
monarch_rare_class <- tax_glom(monarch_rarefy_count, taxrank = "Class")
# plot_bar(monarch_rare_class, fill = 'Class')
monarch_rich_ggplot <- psmelt(monarch_rare_class)

monarch_relabund_class <- tax_glom(monarch_relative, taxrank = "Class")
# plot_bar(monarch_relabund_class, fill = 'Class')
# Pinus_rel_class_rel <-
# transform_sample_counts(Pinus_rel_class, function(x) x /
# sum(x))


monarch_relabund_ggplot <- psmelt(monarch_relabund_class)
order_Asco <- c("Leotiomycetes", "Eurotiomycetes", "Sordariomycetes",
    "Lecanoromycetes", "Pezizomycetes", "Saccharomycetes", "Arthoniomycetes",
    "Orbiliomycetes", "Pezizomycotina incertae sedis", "Sareomycetes",
    "Taphrinomycetes", "Dothideomycetes")
order_Basidio <- c("Agaricomycetes", "Agaricostilbomycetes",
    "Atractiellomycetes", "Cystobasidiomycetes", "Exobasidiomycetes",
    "Malasseziomycetes", "Microbotryomycetes", "Pucciniomycetes",
    "Tremellomycetes", "Ustilaginomycetes", "Wallemiomycetes",
    "Classiculomycetes", "Spiculogloeomycetes")
order_incertae <- c("Chytridiomycetes", "Mucoromycetes")
order_glomer <- c("Glomeromycetes")

monarch_relabund_ggplot$Class <- factor(monarch_relabund_ggplot$Class,
    levels = c("BASIDIOMYCOTA", order_Basidio, "  ", "   ", "    ",
        "     ", "ASCOMYCOTA", order_Asco, " ", "Others", order_incertae,
        order_glomer))

monarch_rich_ggplot$Class <- factor(monarch_rich_ggplot$Class,
    levels = c("BASIDIOMYCOTA", order_Basidio, "  ", "   ", "    ",
        "     ", "ASCOMYCOTA", order_Asco, " ", "Others", order_incertae,
        order_glomer))
```

```r
monarch_relabund_ggplot$material_species = factor(monarch_relabund_ggplot$material_species,
    levels = c("plant_currasavica", "frass_currasavica", "plant_fascicularis",
        "frass_fascicularis"))


monarch_rich_ggplot$material_species = factor(monarch_rich_ggplot$material_species,
    levels = c("plant_currasavica", "frass_currasavica", "plant_fascicularis",
        "frass_fascicularis"))

############################################## 3


barplot(rep(1, 12), col = c("violetred4", "gold1", "salmon1",
    "pink3", "gold3", "darkorange3", "orange2", "wheat3", "tomato3",
    "lightpink", "lavenderblush3", "lightgoldenrod1"))
```

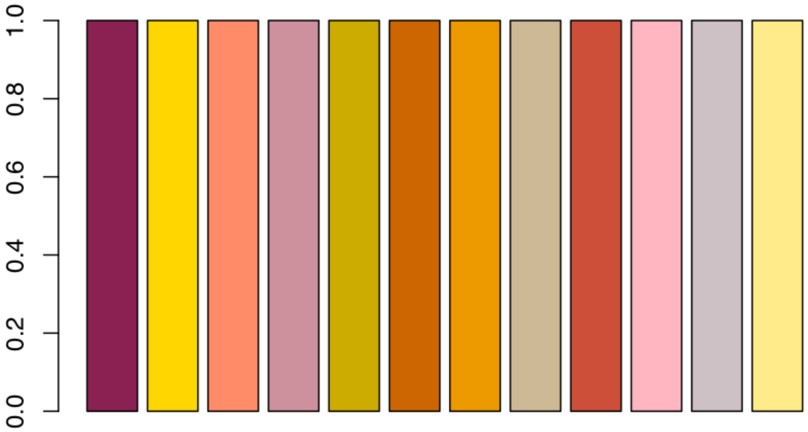


```r
Asco_colors <- c("violetred4", "gold1", "salmon1", "pink3", "lightgoldenrod1",
    "darkorange3", "orange2", "wheat3", "tomato3", "lightpink",
    "lavenderblush3", "gold3")

barplot(rep(1, 13), col = c("#5E738F", "cadetblue3", "slategray3",
    "lightcyan4", "lightsteelblue1", "darkcyan", "paleturquoise",
    "slateblue3", "gray82", "cornflowerblue", "dodgerblue4",
    "paleturquoise4", "plum4"))
```

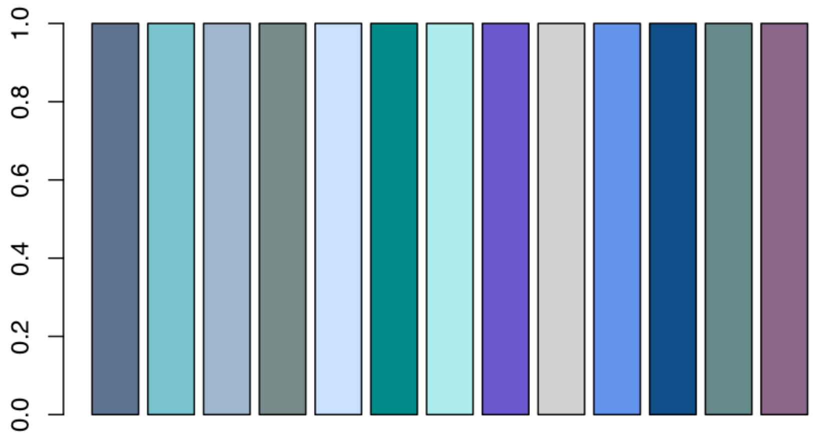


```
Basidio_colors <- c("#5E738F", "cadetblue3", "slategray3", "lightcyan4",
    "lightsteelblue1", "darkcyan", "paleturquoise", "slateblue3",
    "plum4", "cornflowerblue", "dodgerblue4", "paleturquoise4",
    "gray82")

barplot(rep(1, 5), col = c("darkolivegreen", "darkolivegreen3"))
```

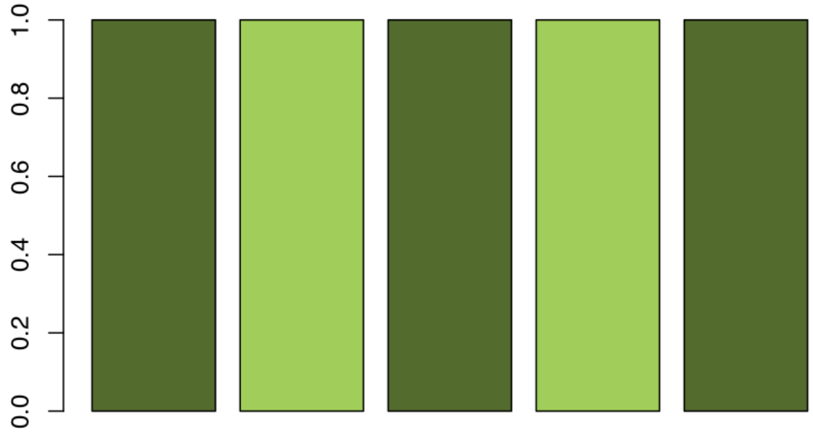


```
Incertae_colors <- c("darkolivegreen", "darkolivegreen3")
glomer_color <- "darkorange1"

trt_names <- as_labeller(c(plant_currasavica = "A. curassavica leaves",
    frass_currasavica = "A. curassavica frass", plant_fascicularis = "A. fascicularis leaves",
    frass_fascicularis = "A. fascicularis frass"))

#######################

monarch_rel_abund_graph <- ggplot(monarch_relabund_ggplot, aes(x = Sample,
    y = Abundance, fill = Class)) + geom_bar(stat = "identity") +
```

```r
    facet_wrap(~material_species, scales = "free_x", nrow = 1,
        labeller = trt_names) + scale_fill_manual(na.translate = FALSE,
    values = c("white", Basidio_colors, "white", "white", "white",
        "white", "white", Asco_colors, "white", "white", Incertae_colors,
        glomer_color), drop = FALSE) + theme_bw() + theme(legend.position = "bottom",
    legend.key = element_rect(fill = NA), legend.title = element_blank(),
    legend.spacing.x = unit(0.2, "cm")) + guides(fill = guide_legend(reverse = FALSE,
    ncol = 4)) + labs(y = "Relative abundance of ASVs by class",
    x = "Samples") + theme(axis.text = element_text(size = 8),
    axis.text.x = element_text(angle = 45, hjust = 1), title = element_text(face = "italic",
        size = 20, hjust = 0), strip.text.x = element_text(size = 15),
    strip.background = element_blank(), axis.title = element_text(face = "plain",
        size = 20), plot.title = element_text(hjust = 0), axis.title.y = element_text(hjust = 0.5,
        margin = margin(0, 10, 0, 0)), panel.border = element_blank(),
    panel.grid.major = element_blank(), panel.grid.minor = element_blank(),
    axis.line = element_line(colour = "black"))

png(here::here("Output", "BarGraph.png"), units = "in", width = 10,
    height = 9, res = 400)
monarch_rel_abund_graph
dev.off()
```

```
## pdf
##   2
```

```r
monarch_rich_graph <- ggplot(monarch_rich_ggplot, aes(x = Sample,
    y = Abundance, fill = Class)) +
geom_bar(stat = "identity") + facet_wrap(~material_species, scales = "free_x",
    nrow = 1, labeller = trt_names) + scale_fill_manual(na.translate = FALSE,
    values = c("white", Basidio_colors, "white", "white", "white",
        "white", "white", Asco_colors, "white", "white", Incertae_colors,
        glomer_color), drop = FALSE) + theme_bw() + theme(legend.position = "bottom",
    legend.key = element_rect(fill = NA), legend.title = element_blank(),
    legend.spacing.x = unit(0.2, "cm")) + guides(fill = guide_legend(reverse = FALSE,
    ncol = 4)) + labs(y = "ASV Richness", x = "Samples") + theme(axis.text = element_text(size = 8),
    axis.text.x = element_text(angle = 45, hjust = 1), title = element_text(face = "italic",
        size = 20, hjust = 0), strip.text.x = element_text(size = 15),
    axis.title = element_text(face = "plain", size = 20), plot.title = element_text(hjust = 0),
    axis.title.y = element_text(hjust = 0.5, margin = margin(0,
        10, 0, 0)), panel.border = element_blank(), panel.grid.major = element_blank(),
    panel.grid.minor = element_blank(), axis.line = element_line(colour = "black"))



png(here::here("Output", "Monarch_richness.png"), units = "in",
    width = 10, height = 8, res = 400)

monarch_rich_graph
dev.off()
```

```
## pdf
##   2
```

```r
######################
```

We'd like to find the taxa subset that best explains the community differences. http://menugget.blogspot.

com/2011/06/clarke-and-ainsworths-bioenv-and-bvstep.html

the bv.step takes a very long time

```
# Monarch_merged_nMDS<- metaMDS(Monarch_merged_BCData,
# distance = 'bray', k=2, trymax = 1000)

cmethod <- "pearson"  #Correlation method to use: pearson, pearman, kendall
fmethod <- "bray"  #Fixed distance method: euclidean, manhattan, gower, altGower, canberra, bray, kulcz
vmethod <- "bray"  #Variable distance method: euclidean, manhattan, gower, altGower, canberra, bray, ku
nmethod <- "bray"  #NMDS distance method:  euclidean, manhattan, gower, altGower, canberra, bray, kulcz

res.bv.step.biobio <- bv.step(Monarch_merged_tCSSmat, Monarch_merged_tCSSmat,
    fix.dist.method = "bray", var.dist.method = "bray", scale.fix = FALSE,
    scale.var = FALSE, max.rho = 0.95, min.delta.rho = 0.001,
    random.selection = TRUE, prop.selected.var = 0.3, num.restarts = 50,
    output.best = 10, var.always.include = NULL)
```

```
## [1] "2 % finished"
## [1] "4 % finished"
## [1] "6 % finished"
## [1] "8 % finished"
## [1] "10 % finished"
## [1] "12 % finished"
## [1] "14 % finished"
## [1] "16 % finished"
## [1] "18 % finished"
## [1] "20 % finished"
## [1] "22 % finished"
## [1] "24 % finished"
## [1] "26 % finished"
## [1] "28 % finished"
## [1] "30 % finished"
## [1] "32 % finished"
## [1] "34 % finished"
## [1] "36 % finished"
## [1] "38 % finished"
## [1] "40 % finished"
## [1] "42 % finished"
## [1] "44 % finished"
## [1] "46 % finished"
## [1] "48 % finished"
## [1] "50 % finished"
## [1] "52 % finished"
## [1] "54 % finished"
## [1] "56 % finished"
## [1] "58 % finished"
## [1] "60 % finished"
## [1] "62 % finished"
## [1] "64 % finished"
## [1] "66 % finished"
## [1] "68 % finished"
## [1] "70 % finished"
## [1] "72 % finished"
## [1] "74 % finished"
```

```
## [1] "76 % finished"
## [1] "78 % finished"
## [1] "80 % finished"
## [1] "82 % finished"
## [1] "84 % finished"
## [1] "86 % finished"
## [1] "88 % finished"
## [1] "90 % finished"
## [1] "92 % finished"
## [1] "94 % finished"
## [1] "96 % finished"
## [1] "98 % finished"
## [1] "100 % finished"
```

```
res.bv.step.biobio$order.by.best
```

```
##                                              var.incl n.var       rho
## 1  1,2,76,109,159,180,190,202,222,238,276,358,613,701    14 0.7824301
## 2      1,2,76,159,180,190,202,222,238,276,358,613,701    13 0.7820774
## 3          1,2,76,159,180,190,222,238,276,358,613,701    12 0.7793610
## 4             1,2,159,180,190,222,238,276,358,613,701    11 0.7746245
## 5                 1,2,159,180,190,238,276,358,613,701    10 0.7696067
## 6                     1,2,159,180,190,238,276,358,613     9 0.7654888
## 7                       2,159,180,190,238,276,358,613     8 0.7602859
## 8                         159,180,190,238,276,358,613     7 0.7503116
## 9                           2,159,180,190,238,358,613     7 0.7492902
## 10                              2,159,190,238,358,613     6 0.7384166
```

```
res.bv.step.biobio$order.by.i.comb
```

```
##                                              var.incl n.var       rho
## 1                                                 190     1 0.5247522
## 2                                             358,613     2 0.6169003
## 3                                         190,358,613     3 0.6823199
## 4                                     159,190,358,613     4 0.7032845
## 5                                 159,190,238,358,613     5 0.7291126
## 6                               2,159,190,238,358,613     6 0.7384166
## 7                         159,180,190,238,276,358,613     7 0.7503116
## 8                       2,159,180,190,238,276,358,613     8 0.7602859
## 9                     1,2,159,180,190,238,276,358,613     9 0.7654888
## 10                1,2,159,180,190,238,276,358,613,701    10 0.7696067
## 11            1,2,159,180,190,222,238,276,358,613,701    11 0.7746245
## 12         1,2,76,159,180,190,222,238,276,358,613,701    12 0.7793610
## 13     1,2,76,159,180,190,202,222,238,276,358,613,701    13 0.7820774
## 14 1,2,76,109,159,180,190,202,222,238,276,358,613,701    14 0.7824301
```

```
res.bv.step.biobio$best.model.vars
```

```
## [1] "Seq1,Seq10,Seq15,Seq19,Seq25,Seq28,Seq29,Seq30,Seq33,Seq35,Seq4,Seq5,Seq8,Seq9"
```

```
res.bv.step.biobio$best.model.rho
```

```
## [1] 0.7824301
```

```
res.bv.step.biobio$var.always.include
```

```
## NULL
```

```r
# res.bv.step.biobio$var.exclude
```

```r
res.bv.step.biobio2 <- bv.step(Monarch_merged_tCSSmat, Monarch_merged_tCSSmat,
    fix.dist.method = "bray", var.dist.method = "bray", scale.fix = FALSE,
    scale.var = FALSE, max.rho = 0.95, min.delta.rho = 0.001,
    random.selection = TRUE, prop.selected.var = 0.2, num.restarts = 50,
    output.best = 10, var.always.include = c(190, 358, 613))
```

```
## [1] "2 % finished"
## [1] "4 % finished"
## [1] "6 % finished"
## [1] "8 % finished"
## [1] "10 % finished"
## [1] "12 % finished"
## [1] "14 % finished"
## [1] "16 % finished"
## [1] "18 % finished"
## [1] "20 % finished"
## [1] "22 % finished"
## [1] "24 % finished"
## [1] "26 % finished"
## [1] "28 % finished"
## [1] "30 % finished"
## [1] "32 % finished"
## [1] "34 % finished"
## [1] "36 % finished"
## [1] "38 % finished"
## [1] "40 % finished"
## [1] "42 % finished"
## [1] "44 % finished"
## [1] "46 % finished"
## [1] "48 % finished"
## [1] "50 % finished"
## [1] "52 % finished"
## [1] "54 % finished"
## [1] "56 % finished"
## [1] "58 % finished"
## [1] "60 % finished"
## [1] "62 % finished"
## [1] "64 % finished"
## [1] "66 % finished"
## [1] "68 % finished"
## [1] "70 % finished"
## [1] "72 % finished"
## [1] "74 % finished"
## [1] "76 % finished"
## [1] "78 % finished"
## [1] "80 % finished"
## [1] "82 % finished"
## [1] "84 % finished"
## [1] "86 % finished"
```

```
## [1] "88 % finished"
## [1] "90 % finished"
## [1] "92 % finished"
## [1] "94 % finished"
## [1] "96 % finished"
## [1] "98 % finished"
## [1] "100 % finished"
```

```r
res.bv.step.biobio2$order.by.best
```

```
##                                              var.incl n.var       rho
## 1  1,2,76,109,159,180,190,202,222,238,276,358,613,701    14 0.7824301
## 2      1,2,76,159,180,190,202,222,238,276,358,613,701    13 0.7820774
## 3          1,2,76,159,180,190,222,238,276,358,613,701    12 0.7793610
## 4             1,2,159,180,190,222,238,276,358,613,701    11 0.7746245
## 5                 1,2,159,180,190,238,276,358,613,701    10 0.7696067
## 6                     1,2,159,180,190,238,276,358,613     9 0.7654888
## 7                       2,159,180,190,238,276,358,613     8 0.7602859
## 8                         159,180,190,238,276,358,613     7 0.7503116
## 9                           2,159,180,190,238,358,613     7 0.7492902
## 10                              2,159,190,238,358,613     6 0.7384166
```

```r
res.bv.step.biobio2$order.by.i.comb
```

```
##                                              var.incl n.var       rho
## 1                                                 190     1 0.5247522
## 2                                             358,613     2 0.6169003
## 3                                         190,358,613     3 0.6823199
## 4                                     159,190,358,613     4 0.7032845
## 5                                 159,190,238,358,613     5 0.7291126
## 6                               2,159,190,238,358,613     6 0.7384166
## 7                         159,180,190,238,276,358,613     7 0.7503116
## 8                       2,159,180,190,238,276,358,613     8 0.7602859
## 9                     1,2,159,180,190,238,276,358,613     9 0.7654888
## 10                1,2,159,180,190,238,276,358,613,701    10 0.7696067
## 11            1,2,159,180,190,222,238,276,358,613,701    11 0.7746245
## 12         1,2,76,159,180,190,222,238,276,358,613,701    12 0.7793610
## 13     1,2,76,159,180,190,202,222,238,276,358,613,701    13 0.7820774
## 14 1,2,76,109,159,180,190,202,222,238,276,358,613,701    14 0.7824301
```

```r
res.bv.step.biobio2$best.model.vars
```

```
## [1] "Seq1,Seq10,Seq15,Seq19,Seq25,Seq28,Seq29,Seq30,Seq33,Seq35,Seq4,Seq5,Seq8,Seq9"
```

```r
res.bv.step.biobio2$best.model.rho
```

```
## [1] 0.7824301
```

```r
res.bv.step.biobio2$var.always.include
```

```
## [1] 190 358 613
```

```r
# res.bv.step.biobio2$var.exclude



bio.keep <- unlist(strsplit(res.bv.step.biobio2$best.model.vars,
    split = ","))
```

```
bio.fit <- envfit(Monarch_merged_nMDS, Monarch_merged_tCSSmat[,
    bio.keep, drop = F], perm = 999)



# Get the vectors for bioenv.fit
df_biofit <- scores(bio.fit, display = c("vectors"))
df_biofit <- df_biofit * vegan:::ordiArrowMul(df_biofit)
df_biofit <- as.data.frame(df_biofit)



for (i in 1:nrow(df_biofit)) {
    if (rownames(df_biofit[i, ]) == "Seq1") {
        df_biofit[i, 3] <- "Vishniacozyma sp."
    } else if (rownames(df_biofit[i, ]) == "Seq10") {
        df_biofit[i, 3] <- "Filobasidium sp."
    } else if (rownames(df_biofit[i, ]) == "Seq15") {
        df_biofit[i, 3] <- "Cladosporium sp. 2"
    } else if (rownames(df_biofit[i, ]) == "Seq19") {
        df_biofit[i, 3] <- "Filobasidium sp."

    } else if (rownames(df_biofit[i, ]) == "Seq25") {
        df_biofit[i, 3] <- "Vishniacozyma sp."
    } else if (rownames(df_biofit[i, ]) == "Seq28") {
        df_biofit[i, 3] <- "Sporobolomyces sp."
    } else if (rownames(df_biofit[i, ]) == "Seq29") {
        df_biofit[i, 3] <- "Cladosporium sp."
    } else if (rownames(df_biofit[i, ]) == "Seq30") {
        df_biofit[i, 3] <- "Vishniacozyma sp."
    } else if (rownames(df_biofit[i, ]) == "Seq33") {
        df_biofit[i, 3] <- "Filobasidium sp."
    } else if (rownames(df_biofit[i, ]) == "Seq35") {
        df_biofit[i, 3] <- "Cladosporium sp."
    } else if (rownames(df_biofit[i, ]) == "Seq4") {
        df_biofit[i, 3] <- "Aureobasidium sp."
    } else if (rownames(df_biofit[i, ]) == "Seq5") {
        df_biofit[i, 3] <- "Vishniacozyma sp."
    } else if (rownames(df_biofit[i, ]) == "Seq8") {
        df_biofit[i, 3] <- "Cladosporium sp."
    } else if (rownames(df_biofit[i, ]) == "Seq9") {
        df_biofit[i, 3] <- "Aureobasidium sp."

    }
}



`%!in%` <- purrr::compose(`!`, `%in%`)
bio.vish <- df_biofit[rownames(df_biofit) %in% c("Seq1", "Seq5",
    "Seq25", "Seq30"), ]
# Seq1 is V. carnescens, Seq5 might be V. heimaeyensis.
bio.filo <- df_biofit[rownames(df_biofit) %in% c("Seq33", "Seq10",
```

```
    "Seq19"), ]
bio.clad <- df_biofit[rownames(df_biofit) %in% c("Seq15", "Seq29",
    "Seq35", "Seq8"), ]
bio.sporo <- df_biofit[rownames(df_biofit) %in% c("Seq28"), ]
bio.aureo <- df_biofit[rownames(df_biofit) %in% c("Seq4", "Seq9"),
    ]

Final_nmds_plot + theme(axis.text = element_text(size = 16),
    axis.title = element_text(size = 21)) + guides(fill = FALSE) +
    geom_segment(data = bio.clad, aes(x = 0, y = 0, xend = NMDS1 *
        0.6, yend = NMDS2 * 0.6), arrow = arrow(length = unit(0.3,
        "cm")), color = "gray31", alpha = 0.5, inherit.aes = FALSE) +
    geom_text(data = bio.clad, aes(NMDS1, NMDS2, label = V3),
        color = "black", alpha = 1, inherit.aes = FALSE) + geom_segment(data = bio.vish,
    aes(x = 0, y = 0, xend = NMDS1 * 0.6, yend = NMDS2 * 0.6),
    arrow = arrow(length = unit(0.3, "cm")), color = "gray31",
    alpha = 0.5, inherit.aes = FALSE) + geom_text(data = bio.vish,
    aes(NMDS1, NMDS2, label = V3), color = "black", alpha = 1,
    inherit.aes = FALSE) + geom_segment(data = bio.sporo, aes(x = 0,
    y = 0, xend = NMDS1 * 0.6, yend = NMDS2 * 0.6), arrow = arrow(length = unit(0.3,
    "cm")), color = "gray31", alpha = 0.5, inherit.aes = FALSE) +
    geom_text(data = bio.sporo, aes(NMDS1, NMDS2, label = V3),
        color = "black", alpha = 1, inherit.aes = FALSE) + geom_segment(data = bio.filo,
    aes(x = 0, y = 0, xend = NMDS1 * 0.6, yend = NMDS2 * 0.6),
    arrow = arrow(length = unit(0.3, "cm")), color = "black",
    alpha = 1, inherit.aes = FALSE) + geom_text(data = bio.filo,
    aes(NMDS1, NMDS2, label = V3), color = "red", alpha = 1,
    inherit.aes = FALSE) + geom_segment(data = bio.aureo, aes(x = 0,
    y = 0, xend = NMDS1 * 0.6, yend = NMDS2 * 0.6), arrow = arrow(length = unit(0.3,
    "cm")), color = "black", alpha = 1, inherit.aes = FALSE) +
    geom_text(data = bio.aureo, aes(NMDS1, NMDS2, label = V3),
        color = "black", alpha = 1, inherit.aes = FALSE)
```

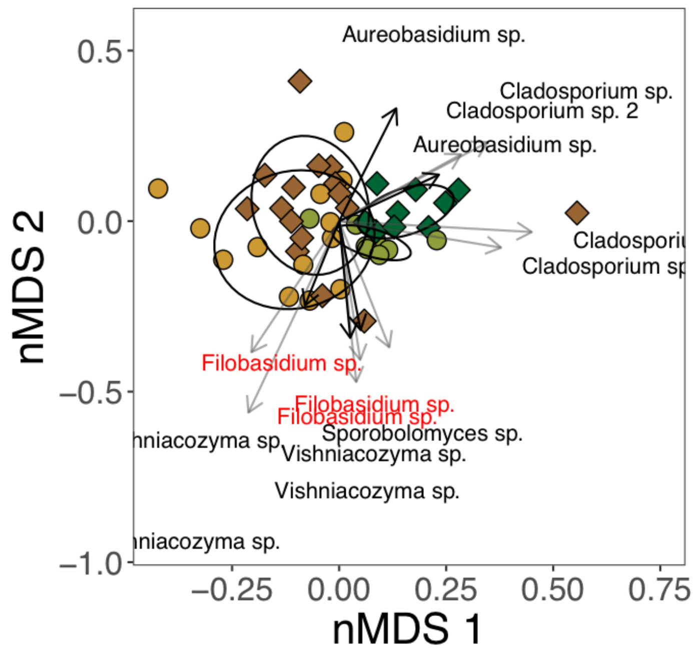


```
NMDS_taxa_vector <- Final_nmds_plot + xlim(-0.45, 0.6) + ylim(-0.6,
    0.411) + theme(axis.text = element_text(size = 16), axis.title = element_text(size = 21)) +
    guides(fill = FALSE) + geom_segment(data = bio.clad, aes(x = 0,
    y = 0, xend = NMDS1 * 0.6, yend = NMDS2 * 0.6), arrow = arrow(length = unit(0.3,
    "cm")), color = "gray31", alpha = 0.5, inherit.aes = FALSE) +
    # geom_text(data=bio.clad, aes(NMDS1*1.1, NMDS2*1.2, label =
# V3), color='black',alpha=1, inherit.aes = FALSE) +
geom_segment(data = bio.vish, aes(x = 0, y = 0, xend = NMDS1 *
    0.6, yend = NMDS2 * 0.6), arrow = arrow(length = unit(0.3,
    "cm")), color = "gray31", alpha = 0.5, inherit.aes = FALSE) +
    # geom_text(data=bio.vish, aes(NMDS1, NMDS2, label = V3),
# color='black',alpha=1, inherit.aes = FALSE) +
geom_segment(data = bio.sporo, aes(x = 0, y = 0, xend = NMDS1 *
    0.6, yend = NMDS2 * 0.6), arrow = arrow(length = unit(0.3,
    "cm")), color = "gray31", alpha = 0.5, inherit.aes = FALSE) +
    # geom_text(data=bio.sporo, aes(NMDS1+0.23, NMDS2*1.1, label
# = V3), color='black',alpha=1, inherit.aes = FALSE) +
geom_segment(data = bio.filo, aes(x = 0, y = 0, xend = NMDS1 *
    0.6, yend = NMDS2 * 0.6), arrow = arrow(length = unit(0.3,
    "cm")), color = "gray31", alpha = 0.5, inherit.aes = FALSE) +
    # geom_text(data=bio.filo, aes(NMDS1-0.15, NMDS2*1.05, label
# = V3), color='black',alpha=1,inherit.aes = FALSE)+
geom_segment(data = bio.aureo, aes(x = 0, y = 0, xend = NMDS1 *
    0.6, yend = NMDS2 * 0.6), arrow = arrow(length = unit(0.3,
    "cm")), color = "gray31", alpha = 0.5, inherit.aes = FALSE)
# geom_text(data=bio.aureo, aes(NMDS1*1.3, NMDS2*1.1, label =
# V3), color='gray31',alpha=0.5,inherit.aes = FALSE)
```

```
NMDS_taxa_vector
```

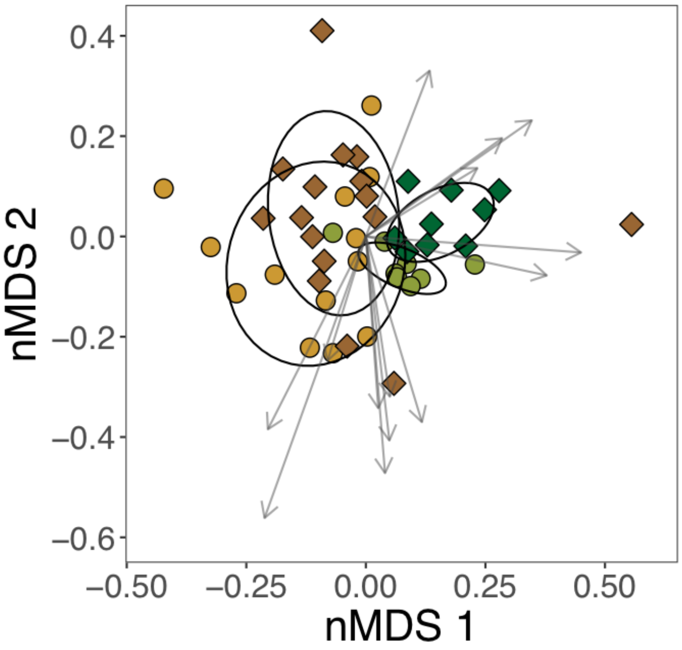


```
png(here::here("Output", "Monarch_nmds_taxa.png"), units = "in",
    width = 7, height = 7, res = 300)
NMDS_taxa_vector
dev.off()
```

```
## pdf
##   2
```

```
fasc_leaf_mat<-phylo_fasc_leaf %>%
  transform_sample_counts(function(x) x / sum(x)) %>%
  otu_table() %>%
  as("matrix")

fasc_leaf_sum<-rbind(colMeans(fasc_leaf_mat),fasc_leaf_mat)
fasc_leaf_sum_ord<-fasc_leaf_sum[,order(fasc_leaf_sum[1,],decreasing=TRUE)]
fasc_leaf_top10<-colnames(fasc_leaf_sum_ord[,1:10])

curr_leaf_mat<-phylo_curr_leaf %>%
  transform_sample_counts(function(x) x / sum(x)) %>%
  otu_table() %>%
  as("matrix")

curr_leaf_sum<-rbind(colMeans(curr_leaf_mat),curr_leaf_mat)
curr_leaf_sum_ord<-curr_leaf_sum[,order(curr_leaf_sum[1,],decreasing=TRUE)]
curr_leaf_top10<-colnames(curr_leaf_sum_ord[,1:10])
```

```r
fasc_frass_mat<-phylo_fasc_frass_per_caterpillar_rare %>%
  transform_sample_counts(function(x) x / sum(x)) %>%
  otu_table() %>%
  as("matrix")

fasc_frass_sum<-rbind(colMeans(fasc_frass_mat),fasc_frass_mat)
fasc_frass_sum_ord<-fasc_frass_sum[,order(fasc_frass_sum[1,],decreasing=TRUE)]
fasc_frass_top10<-colnames(fasc_frass_sum_ord[,1:10])

curr_frass_mat<-phylo_curr_frass_per_caterpillar_rare %>%
  transform_sample_counts(function(x) x / sum(x)) %>%
  otu_table() %>%
  as("matrix")

curr_frass_sum<-rbind(colMeans(curr_frass_mat),curr_frass_mat)
curr_frass_sum_ord<-curr_frass_sum[,order(curr_frass_sum[1,],decreasing=TRUE)]
curr_frass_top10<-colnames(curr_frass_sum_ord[,1:10])

union<-union(fasc_leaf_top10, curr_leaf_top10) %>%
        union(fasc_frass_top10) %>%
        union(curr_frass_top10)

curr_frass<-curr_frass_mat[,colnames(curr_frass_mat) %in% union]
mat.to.add = matrix(0, nrow = dim(curr_frass)[1],
                    ncol = length(setdiff(union,colnames(curr_frass))))
colnames(mat.to.add) <-setdiff(union,colnames(curr_frass))
curr_frass_01 = cbind(curr_frass, mat.to.add)

fasc_frass<-fasc_frass_mat[,colnames(fasc_frass_mat) %in% union]
mat.to.add = matrix(0, nrow = dim(fasc_frass)[1],
                    ncol = length(setdiff(union,colnames(fasc_frass))))
colnames(mat.to.add) <-setdiff(union,colnames(fasc_frass))
fasc_frass_01 = cbind(fasc_frass, mat.to.add)

curr_leaf<-curr_leaf_mat[,colnames(curr_leaf_mat) %in% union]
fasc_leaf<-fasc_leaf_mat[,colnames(fasc_leaf_mat) %in% union]


Rel_abund_table_01 <-rbind(curr_leaf, curr_frass_01[, colnames(curr_leaf)])
Rel_abund_table_02 <- rbind(Rel_abund_table_01,fasc_leaf)
Rel_abund_table_03 <- rbind(Rel_abund_table_02,fasc_frass_01)

#Order the ASVs by class and genus
sub_phyloseq_03<-prune_taxa(union,phyloseq_03)

pruned_pref = apply(X = t(otu_table(sub_phyloseq_03)),
                    MARGIN = 1,
                    FUN = function(x){sum(x > 0)})


sub_phyloseq_03_tax<-as.data.frame(tax_table(sub_phyloseq_03))
sub_phyloseq_03_tax_01 <- sub_phyloseq_03_tax[order(sub_phyloseq_03_tax$Phylum, sub_phyloseq_03_tax$Cla
```

```
#Order of hierarchical cluster
Rel_abund_table_04 <- Rel_abund_table_03[,row.names(sub_phyloseq_03_tax_01)]


abund.melt<- melt(Rel_abund_table_04, id="OTU", value.name="RelativeAbundance", na.rm = TRUE)

abund.melt[, "RelativeAbundance"] %>% sapply(as.character) %>% sapply(as.numeric)


abund.melt <-plyr::ddply(abund.melt, plyr::.(Var1), transform, rescale = rescale(RelativeAbundance)) #t

abund.melt$Var2 <- factor(abund.melt$Var2, levels = row.names(sub_phyloseq_03_tax_01))
abund.melt$Var1 <- factor(abund.melt$Var1, levels = row.names(Rel_abund_table_04))

#Change 'Seq' to 'ASV'
abund.melt$Var2<- as.factor(str_replace_all(abund.melt$Var2, "Seq", "ASV"))
order<- str_replace_all(row.names(sub_phyloseq_03_tax_01), "Seq", "ASV")
abund.melt$Var2 <- factor(abund.melt$Var2, levels = rev(order))


Monarch_heatmap<-ggplot(abund.melt, aes(Var1, Var2, fill=rescale)) +
  geom_tile() + #rectangles for each correlation
  #add actual correlation value in the rectangle
  geom_text(aes(label = round(RelativeAbundance*100, 1)), size = 2.5) +
  theme_bw(base_size=10) + #black and white theme with set font size
  #rotate x-axis labels so they don't overlap,
  #get rid of unnecessary axis titles
  #adjust plot margins
  scale_x_discrete(position = "bottom") +
  theme(plot.margin = unit(c(10, 10, 10, 10), "mm"),
        axis.text.x= element_blank(),
        axis.text.y = element_text(size = 12), text = element_text(size=15),
        legend.position='none') +
  labs(x ="", y ="") +
  #set correlation fill gradient
  scale_fill_gradientn(colours=c("white","lightcyan", "cadetblue3"),
                       limit = c(0,1), space = "Lab",
                       name="Relative\nAbundance\n",
                       values  = c(0, 0.1, 1))
```

```
Monarch_heatmap
```

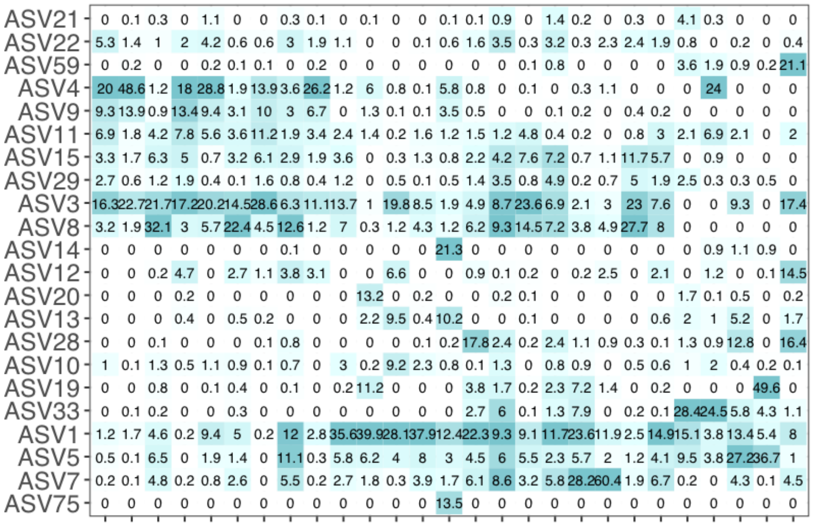


```
png(here::here("Output", "Heatmap1.png"), units = "in", width = 10,
    height = 10, res = 300)
Monarch_heatmap
dev.off()
```

```
## pdf
##   2
```

We will not use indicator species analysis. https://cran.r-project.org/web/packages/indicspecies/vignettes/indicspeciesTutorial.pdf

```
indicator_sp_list <- union(bio.keep, union)

indicator_rare_25 <- prune_taxa(indicator_sp_list, indicator_rare_phylo)

prevalencedf = apply(X = t(otu_table(indicator_rare_25)), MARGIN = 1,
    FUN = function(x) {
        sum(x > 0)
    })

indicator_multi = multipatt(otu_table(indicator_rare_25), sample_data(indicator_rare_25)$material_speci
    control = how(nperm = 99999), max.order = 2)  #sometimes you have to 'transform' the otu table

summary(indicator_multi)
```

```
##
##  Multilevel pattern analysis
##  ---------------------------
##
##  Association function: IndVal.g
##  Significance level (alpha): 0.05
##
##  Total number of species: 25
##  Selected number of species: 9
```

```
##  Number of species associated to 1 group: 0
##  Number of species associated to 2 groups: 9
##  Number of species associated to 3 groups: 0
##
##  List of species associated to each combination:
##
##  Group frass_curassavica+frass_fascicularis  #sps.  4
##        stat p.value
## Seq10 0.963 0.00656 **
## Seq5  0.941 0.00052 ***
## Seq1  0.936 0.00111 **
## Seq13 0.890 0.00547 **
##
##  Group frass_fascicularis+plant_currasavica  #sps.  1
##       stat p.value
## Seq9 0.885  0.0197 *
##
##  Group frass_fascicularis+plant_fascicularis  #sps.  3
##        stat p.value
## Seq7  0.928 0.03004 *
## Seq33 0.913 0.00081 ***
## Seq28 0.884 0.02020 *
##
##  Group plant_currasavica+plant_fascicularis  #sps.  1
##        stat p.value
## Seq35 0.875  0.0127 *
## ---
## Signif. codes:  0 '***' 0.001 '**' 0.01 '*' 0.05 '.' 0.1 ' ' 1
```

```
indicator_multi$sign$p.value.bh <- p.adjust(indicator_multi$sign$p.value,
    method = "BH")

multipatt_25 <- cbind(indicator_multi$str, indicator_multi$sign,
    indicator_multi$A, indicator_multi$B, prevalencedf)
write.csv(multipatt_25, file = here::here("Output", "Monarch_indicator_25_new.csv"))
```

```
install.packages("gt")
```

```
##
## The downloaded binary packages are in
##  /var/folders/m0/49zpc60d1gs_x52krdmr9cdr0000gn/T//RtmpGYFzml/downloaded_packages
```

```
install.packages("webshot")
```

```
##
## The downloaded binary packages are in
##  /var/folders/m0/49zpc60d1gs_x52krdmr9cdr0000gn/T//RtmpGYFzml/downloaded_packages
```

```
library(webshot)
webshot::install_phantomjs()
```

```
## It seems that the version of `phantomjs` installed is greater than or equal to the requested version
```

```
library(gt)
library(tidyverse)
```

```
## -- Attaching packages --------------------------------------- tidyverse 1.3.0 --
```

```
## v tibble  3.0.4     v readr   1.4.0
## v tidyr   1.1.2     v forcats 0.5.0

## -- Conflicts ------------------------------------------ tidyverse_conflicts() --
## x readr::col_factor() masks scales::col_factor()
## x dplyr::combine()    masks Biobase::combine(), BiocGenerics::combine()
## x purrr::discard()    masks scales::discard()
## x tidyr::expand()     masks Matrix::expand()
## x tidyr::extract()    masks magrittr::extract()
## x dplyr::filter()     masks stats::filter()
## x dplyr::lag()        masks stats::lag()
## x tidyr::pack()       masks Matrix::pack()
## x ggplot2::Position() masks BiocGenerics::Position(), base::Position()
## x purrr::set_names()  masks magrittr::set_names()
## x tidyr::unpack()     masks Matrix::unpack()
```

```
library(glue)
```

```
##
## Attaching package: 'glue'

## The following object is masked from 'package:dplyr':
##
##     collapse
```

```
multipatt_25<- read.csv(here::here("Output", "Monarch_indicator_25_new.csv"), header=TRUE, row.names=1,

drop <- c("frass_curassavica","frass_fascicularis","plant_currasavica", "plant_fascicularis",
          "frass_curassavica.plant_fascicularis", "frass_fascicularis.plant_currasavica",
          "frass_curassavica.1","frass_fascicularis.1","plant_currasavica.1", "plant_fascicularis.1",
          "frass_curassavica.plant_fascicularis.1", "frass_fascicularis.plant_currasavica.1",
          "frass_curassavica.2","frass_fascicularis.2","plant_currasavica.2", "plant_fascicularis.2",
          "frass_curassavica.plant_fascicularis.2", "frass_fascicularis.plant_currasavica.2")

taxa.subset <- taxa_table[rownames(multipatt_25), "Genus"]


multipatt_25.v1 = multipatt_25[,!(names(multipatt_25) %in% drop)]
multipatt_25.v2 = merge(taxa.subset,multipatt_25.v1, by=0)
rownames(multipatt_25.v2) <- multipatt_25.v2[,1]
multipatt_25.v2 <- multipatt_25.v2[,-1]

fmt_if_number <- function(..., digits = 2) {
  input <- c(...)
  fmt <- paste0("%.", digits, "f")
  if (is.numeric(input))   return(sprintf(fmt, input))
  return(input)
}


multipatt_table <- multipatt_25.v2 %>%
  arrange(p.value.bh) %>%
    tibble::rownames_to_column() %>%
  gt() %>%
   tab_spanner(
    label = "Specificity",
```

```
    columns = c(
      frass_curassavica.frass_fascicularis.1,
      plant_currasavica.plant_fascicularis.1,
      frass_fascicularis.plant_fascicularis.1,
      frass_curassavica.plant_currasavica.1)
  ) %>%
     tab_spanner(
    label = "Indicator value",
    columns = c(
      frass_curassavica.frass_fascicularis,
      plant_currasavica.plant_fascicularis,
      frass_fascicularis.plant_fascicularis,
      frass_curassavica.plant_currasavica)
  ) %>%
  cols_label(frass_curassavica.frass_fascicularis = "Frass only",
    plant_currasavica.plant_fascicularis = "Leaves only",
    frass_curassavica.plant_currasavica = "A. currasavica only",
    frass_fascicularis.plant_fascicularis = "A. fascicularis only",
    frass_curassavica.frass_fascicularis.1 = "Frass only",
    plant_currasavica.plant_fascicularis.1 = "Leaves only",
    frass_curassavica.plant_currasavica.1 = "A. currasavica only",
    frass_fascicularis.plant_fascicularis.1 = "A. fascicularis only",
    frass_curassavica.frass_fascicularis.2 = "Frass only",
    plant_currasavica.plant_fascicularis.2 = "Leaves only",
    frass_curassavica.plant_currasavica.2= "A. currasavica only",
    frass_fascicularis.plant_fascicularis.2 = "**A. fascicularis only",
        frass_fascicularis.plant_fascicularis.2 = "A. fascicularis only",
    p.value.bh = "BH-corrected p-value",
    p.value = "p-value",
    x = "Genus",
    ) %>%
 tab_spanner(
    label = "Fidelity",
    columns = c(
      frass_curassavica.frass_fascicularis.2,
      plant_currasavica.plant_fascicularis.2,
      frass_fascicularis.plant_fascicularis.2,
      frass_curassavica.plant_currasavica.2)
  )  %>%
  tab_style(
    style = list(
      cell_text(weight = "bold")
      ),
    locations = cells_body(
      columns = vars(frass_curassavica.frass_fascicularis),
      rows = frass_curassavica.frass_fascicularis == stat
    )
  ) %>%
  opt_row_striping() %>%
   tab_style(
    style = list(
      cell_text(weight = "bold")
      ),
```

```
    locations = cells_body(
      columns = vars(plant_currasavica.plant_fascicularis),
      rows = plant_currasavica.plant_fascicularis == stat
    )
  ) %>%
   tab_style(
    style = list(
      cell_text(weight = "bold")
      ),
    locations = cells_body(
      columns = vars(frass_fascicularis.plant_fascicularis),
      rows = frass_fascicularis.plant_fascicularis == stat
    )
  ) %>%
   fmt(everything(),
    fns = function(x)
    fmt_if_number(x, digits = 3,
                  drop_trailing_zeros = TRUE)


    )%>%
  cols_hide(
    columns = c(
      s.frass_curassavica,
      s.frass_fascicularis,
      s.plant_currasavica,
      s.plant_fascicularis,
      prevalencedf, stat, index,
    )
  )  %>% cols_move(
    columns = p.value,
    after = prevalencedf
    ) %>%
  cols_move(
    columns = p.value.bh,
    after = p.value
    )


multipatt_table
```

		Indicator value				
	Genus	Frass only	Leaves only	A. fascicularis only	A. currasavica only	Frass only
Seq1	Vishniacozyma	0.936	0.353	0.495	0.869	0.876
Seq33	Filobasidium	0.396	0.598	0.913	0.057	0.392
Seq5	Vishniacozyma	0.941	0.318	0.785	0.573	0.885
Seq10	Filobasidium	0.963	0.246	0.396	0.870	0.927
Seq13	Unknown Atractiellomycetes	0.890	0.052	0.406	0.571	0.991
Seq35	Cladosporium	0.335	0.875	0.698	0.560	0.187
Seq28	Sporobolomyces	0.382	0.677	0.884	0.166	0.292
Seq9	Aureobasidium	0.660	0.652	0.492	0.806	0.484
Seq7	Vishniacozyma	0.856	0.502	0.928	0.360	0.732
Seq30	Vishniacozyma	0.848	0.232	0.701	0.407	0.899

Seq4	Aureobasidium	0.660	0.590	0.464	0.774	0.545
Seq25	Vishniacozyma	0.808	0.422	0.873	0.252	0.725
Seq22	Alternaria	0.518	0.815	0.631	0.727	0.336
Seq19	Filobasidium	0.814	0.414	0.616	0.595	0.735
Seq29	Cladosporium	0.513	0.790	0.665	0.666	0.376
Seq11	Cladosporium	0.752	0.592	0.638	0.720	0.628
Seq20	Candida	0.545	0.040	0.138	0.443	0.991
Seq3	Cladosporium	0.771	0.582	0.573	0.803	0.661
Seq75	Mucor	0.316	0.000	0.000	0.267	1.000
Seq59	Alternaria	0.545	0.061	0.553	0.044	0.991
Seq14	Preussia	0.316	0.007	0.000	0.378	0.999
Seq12	Unknown Dothideomycetes	0.259	0.482	0.214	0.671	0.671
Seq15	Cladosporium	0.652	0.684	0.717	0.641	0.532
Seq21	Alternaria	0.697	0.128	0.727	0.104	0.972
Seq8	Cladosporium	0.749	0.663	0.755	0.656	0.560

```
multipatt_table %>%
  gtsave(
    "tab_1.pdf",
    path = here::here("Output","Table.pdf")
  )
```

		Indicator value				Specificity				Fidelity					
	Genus	Frass only	Leaves only	A. fascicularis only	A. currasavica only	Frass only	Leaves only	A. fascicularis only	A. currasavica only	Frass only	Leaves only	A. fascicularis only	A. currasavica only	p-value	BH-corrected p-value
Seq1	Vishniacozyma	**0.936**	0.353	0.495	0.869	0.876	0.124	0.245	0.755	1.000	1.000	1.000	1.000	0.001	0.009
Seq33	Filobasidium	0.396	0.598	**0.913**	0.057	0.392	0.608	0.985	0.015	0.400	0.588	0.846	0.214	0.001	0.009
Seq5	Vishniacozyma	**0.941**	0.318	0.785	0.573	0.885	0.115	0.616	0.384	1.000	0.882	1.000	0.857	0.001	0.009
Seq10	Filobasidium	**0.963**	0.246	0.396	0.870	0.927	0.073	0.186	0.814	1.000	0.824	0.846	0.929	0.007	0.033
Seq13	Unknown Atractiellomycetes	**0.890**	0.052	0.406	0.571	0.991	0.009	0.429	0.571	0.800	0.294	0.385	0.571	0.005	0.033
Seq35	Cladosporium	0.335	**0.875**	0.698	0.560	0.187	0.813	0.634	0.366	0.600	0.941	0.769	0.857	0.013	0.053
Seq28	Sporobolomyces	0.382	0.677	**0.884**	0.166	0.292	0.708	0.923	0.077	0.500	0.647	0.846	0.357	0.020	0.063
Seq9	Aureobasidium	0.660	0.652	0.492	0.806	0.484	0.516	0.350	0.650	0.900	0.824	0.692	1.000	0.020	0.063
Seq7	Vishniacozyma	0.856	0.502	**0.928**	0.360	0.732	0.268	0.860	0.140	1.000	0.941	1.000	0.929	0.030	0.083
Seq30	Vishniacozyma	**0.848**	0.232	0.701	0.407	0.899	0.101	0.710	0.290	0.800	0.529	0.692	0.571	0.070	0.158
Seq4	Aureobasidium	0.660	0.590	0.464	0.774	0.545	0.455	0.401	0.599	0.800	0.765	0.538	1.000	0.066	0.158
Seq25	Vishniacozyma	0.808	0.422	**0.873**	0.252	0.725	0.275	0.901	0.099	0.900	0.647	0.846	0.643	0.080	0.166
Seq22	Alternaria	0.518	**0.815**	0.631	0.727	0.336	0.664	0.431	0.569	0.800	1.000	0.923	0.929	0.103	0.199
Seq19	Filobasidium	0.814	0.414	0.616	0.595	0.735	0.265	0.449	0.551	0.900	0.647	0.846	0.643	0.155	0.276
Seq29	Cladosporium	0.513	0.790	0.665	0.666	0.376	0.624	0.522	0.478	0.700	1.000	0.846	0.929	0.194	0.323
Seq11	Cladosporium	0.752	0.592	0.638	0.720	0.628	0.372	0.481	0.519	0.900	0.941	0.846	1.000	0.352	0.487
Seq20	Candida	0.545	0.040	0.138	0.443	0.991	0.009	0.083	0.917	0.300	0.176	0.231	0.214	0.351	0.487
Seq3	Cladosporium	0.771	0.582	0.573	0.803	0.661	0.339	0.356	0.644	0.900	1.000	0.923	1.000	0.314	0.487
Seq75	Mucor	0.316	0.000	0.000	0.267	1.000	0.000	0.000	1.000	0.100	0.000	0.000	0.071	0.370	0.487
Seq59	Alternaria	0.545	0.061	0.553	0.044	0.991	0.009	0.996	0.004	0.300	0.412	0.308	0.429	0.418	0.522
Seq14	Preussia	0.316	0.007	0.000	0.378	0.999	0.001	0.000	1.000	0.100	0.059	0.000	0.143	0.439	0.522
Seq12	Unknown Dothideomycetes	0.259	0.482	0.214	0.671	0.671	0.329	0.099	0.901	0.100	0.706	0.462	0.500	0.597	0.678
Seq15	Cladosporium	0.652	0.684	**0.717**	0.641	0.532	0.468	0.558	0.442	0.800	1.000	0.923	0.929	0.939	0.959
Seq21	Alternaria	0.697	0.128	**0.727**	0.104	0.972	0.028	0.981	0.019	0.500	0.588	0.538	0.571	0.921	0.959
Seq8	Cladosporium	0.749	0.663	**0.755**	0.656	0.560	0.440	0.570	0.430	1.000	1.000	1.000	1.000	0.959	0.959

```
multipatt_table %>%
  gtsave(
    "tab_1.png", vwidth = 2000,
    path = here::here("Output")
  )
```

		Indicator value				Specificity				Fidelity					
	Genus	Frass only	Leaves only	A. fascicularis only	A. currasavica only	Frass only	Leaves only	A. fascicularis only	A. currasavica only	Frass only	Leaves only	A. fascicularis only	A. currasavica only	p-value	BH-corrected p-value
Seq1	Vishniacozyma	**0.936**	0.353	0.495	0.869	0.876	0.124	0.245	0.755	1.000	1.000	1.000	1.000	0.001	0.009
Seq33	Filobasidium	0.396	0.598	**0.913**	0.057	0.392	0.608	0.985	0.015	0.400	0.588	0.846	0.214	0.001	0.009
Seq5	Vishniacozyma	**0.941**	0.318	0.785	0.573	0.885	0.115	0.616	0.384	1.000	0.882	1.000	0.857	0.001	0.009
Seq10	Filobasidium	**0.963**	0.246	0.396	0.870	0.927	0.073	0.186	0.814	1.000	0.824	0.846	0.929	0.007	0.033
Seq13	Unknown Atractiellomycetes	**0.890**	0.052	0.406	0.571	0.991	0.009	0.429	0.571	0.800	0.294	0.385	0.571	0.005	0.033
Seq35	Cladosporium	0.335	**0.876**	0.698	0.560	0.187	0.813	0.634	0.366	0.600	0.941	0.769	0.857	0.013	0.053
Seq28	Sporobolomyces	0.382	0.677	**0.884**	0.166	0.292	0.708	0.923	0.077	0.500	0.647	0.846	0.357	0.020	0.063
Seq9	Aureobasidium	0.660	0.652	0.492	0.806	0.484	0.516	0.350	0.650	0.900	0.824	0.692	1.000	0.020	0.063
Seq7	Vishniacozyma	0.856	0.502	**0.928**	0.360	0.732	0.268	0.860	0.140	1.000	0.941	1.000	0.929	0.030	0.083
Seq30	Vishniacozyma	**0.848**	0.232	0.701	0.407	0.899	0.101	0.710	0.290	0.800	0.529	0.692	0.571	0.070	0.158
Seq4	Aureobasidium	0.660	0.590	0.464	0.774	0.545	0.455	0.401	0.599	0.800	0.765	0.538	1.000	0.066	0.158
Seq25	Vishniacozyma	0.808	0.422	**0.873**	0.252	0.725	0.275	0.901	0.099	0.900	0.647	0.846	0.643	0.080	0.166
Seq22	Alternaria	0.518	**0.815**	0.631	0.727	0.336	0.664	0.431	0.569	0.800	1.000	0.923	0.929	0.103	0.199
Seq19	Filobasidium	0.814	0.414	0.616	0.595	0.735	0.265	0.449	0.551	0.900	0.647	0.846	0.643	0.155	0.276
Seq29	Cladosporium	0.513	0.790	0.665	0.666	0.376	0.624	0.522	0.478	0.700	1.000	0.846	0.929	0.194	0.323
Seq11	Cladosporium	0.752	0.592	0.638	0.720	0.628	0.372	0.481	0.519	0.900	0.941	0.846	1.000	0.352	0.487
Seq20	Candida	0.545	0.040	0.138	0.443	0.991	0.009	0.083	0.917	0.300	0.176	0.231	0.214	0.351	0.487
Seq3	Cladosporium	0.771	0.582	0.573	0.803	0.661	0.339	0.356	0.644	0.900	1.000	0.923	1.000	0.314	0.487
Seq75	Mucor	0.316	0.000	0.000	0.267	1.000	0.000	0.000	1.000	0.100	0.000	0.000	0.071	0.370	0.487
Seq59	Alternaria	0.545	0.061	0.553	0.044	0.991	0.009	0.996	0.004	0.300	0.412	0.308	0.429	0.418	0.522
Seq14	Preussia	0.316	0.007	0.000	0.378	0.999	0.001	0.000	1.000	0.100	0.059	0.000	0.143	0.439	0.522
Seq12	Unknown Dothideomycetes	0.259	0.482	0.214	0.671	0.671	0.329	0.099	0.901	0.100	0.706	0.462	0.500	0.597	0.678
Seq15	Cladosporium	0.652	0.684	**0.717**	0.641	0.532	0.468	0.558	0.442	0.800	1.000	0.923	0.929	0.939	0.959
Seq21	Alternaria	0.697	0.128	**0.727**	0.104	0.972	0.028	0.981	0.019	0.500	0.588	0.538	0.571	0.921	0.959
Seq8	Cladosporium	0.749	0.663	**0.755**	0.656	0.560	0.440	0.570	0.430	1.000	1.000	1.000	1.000	0.959	0.959
