# Supplementary figures and images for: How do phytophagous insects affect phyllosphere fungi? Tracking fungi from milkweed to monarch caterpillar frass reveals communities dominated by fungal yeast
Source: Environ Microbiol Rep. 2024 May 13;16(3):e13213. doi: 10.1111/1758-2229.13213 (PMC11089944; doi:10.1111/1758-2229.13213)

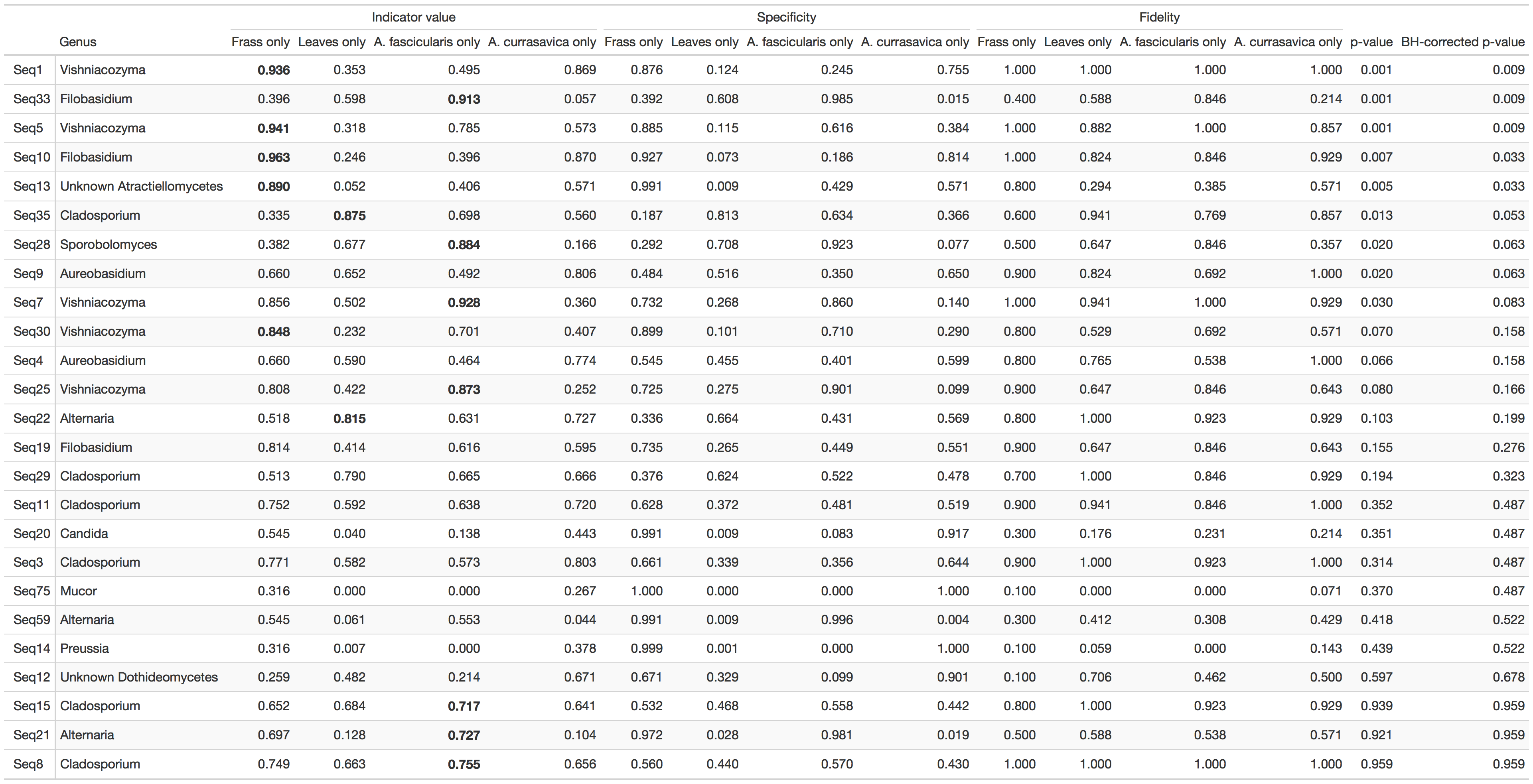

Supplement: Supplementary file 5 — APPENDIX S5. Results of indicator species analysis. [file EMI4-16-e13213-s006.png]

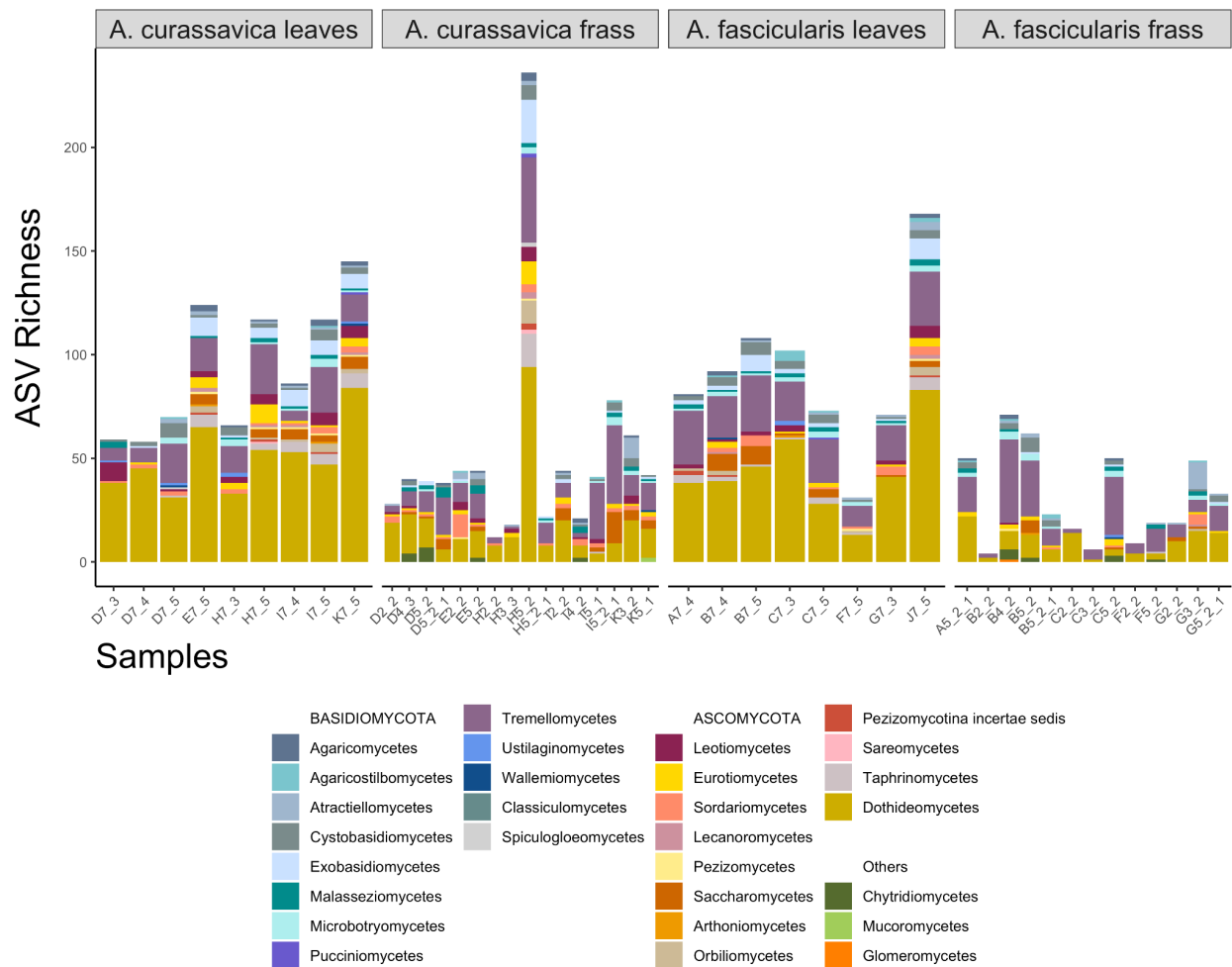

**Appendix S7. Observed ASV richness from rarefied community (1728 reads per sample).**

Supplement: Supplementary file 7 — APPENDIX S7. Observed ASV richness from the rarefied community (1728 reads per sample). [file EMI4-16-e13213-s007.pdf]
