# Supplementary material for: How do phytophagous insects affect phyllosphere fungi? Tracking fungi from milkweed to monarch caterpillar frass reveals communities dominated by fungal yeast
Source: Environ Microbiol Rep. 2024 May 13;16(3):e13213. doi: 10.1111/1758-2229.13213 (PMC11089944; doi:10.1111/1758-2229.13213)
Supplement: Supplementary file 6 — APPENDIX S6. Top BLAST hits of ASVs mentioned in this study. [file EMI4-16-e13213-s008.pdf]

ASV#	Species	Nearest Genbank Accession Name	Nearest GenBank Ascension #	Percent Identity	#bp difference	Source according to GenBank
1	Vishniacozyma sp. 1	Vishniacozyma carnescens CBS:8393	KY105819.1	100%	0	Plant
3	Cladosporium sp. 4	Cladosporium delicatulum	MT548673.1	100%	0	Seed of Danthonia californica
4	Aureobasidium sp. 1	Aureobasidium pullulans	MT573468.1	99.61%	1	Leaf of Rubus idaeus
5	Vishniacozyma sp. 2	Vishniacozyma heimaeyensis CBS:9025	KY105824.1	99.54%	1	Plant
7	Vishniacozyma sp. 3	Vishniacozyma carnescens CBS:8393	KY105819.1	99.53%	1	Plant
8	Cladosporium sp. 5	Cladosporium pseudocladosporioides	OP596073.1	100%	0	Glycine max (soybean)
9	Aureobasidium sp. 2	Aureobasidium pullulans	MT573468.1	100%	0	Leaf of Rubus idaeus
10	Filobasidium sp. 1	Filobasidium magnum	MF964293.1	100%	0	Cotton boll
11	Cladosporium sp. 1	Cladosporium aphidis	MT852116.1	100%	0	Plant
12	Dothideomycetes sp. 5	Pseudophaeophleospora atkinsonii	GU214643.1	92.48%	17	Leaf of Hebe sp.
13	Atractiellomycetes sp. 1	Atractidochium hillariae	MF461287.1	100%	0	Pine needle
14	Preussia sp. 1	Preussia africana	OM744792.1	99.10%	2	Plant
15	Cladosporium sp. 2	Cladosporium ramotenellum	MT441591.1	100%	0	Aristolochia chilensis root tissues
19	Filobasidium sp. 2	Filobasidium floriforme	OM744908.1	100%	0	Plant
20	Candida sp. 1	Candida intermedia	KM246246.1	100%	0	Plant
21	Alternaria sp. 1	Alternaria sorghi	MN534788.1	100.00%	0	Wheat
22	Alternaria sp. 2	Alternaria tenuissima	MT573466.1	100.00%	0	Leaf of Rubus idaeus
25	Vishniacozyma sp. 4	Vishniacozyma victoriae	HG994926.1	97.70%	5	Green bean seeds
28	Sporobolomyces sp. 1	Sporobolomyces lactucae	OK624451.1	100%	0	Phylloplane
29	Cladosporium sp. 3	Cladosporium tenellum	MK513836.1	100%	0	Limestone
30	Vishniacozyma sp. 5	Vishniacozyma heimaeyensis CBS:9025	KY105824.1	100%	0	Plant
33	Filobasidium sp. 3	Filobasidium floriforme CBS:6240	KY103416.1	100%	0	Plant
35	Cladosporium sp. 6	Cladosporium allicinum	MT573471.1	100%	0	Leaf of Rubus idaeus
59	Alternaria sp. 3	Alternaria infectoria	MT561399.1	98.90%	3	Wood of Fagus sylvatica
75	Mucor sp. 1	Mucro racemosus	MT530270.1	100%	0	Unknown

Appendix S6. Top BLAST hits of ASVs specifically used in indicator species analyses or nmds vector analyses
