# Supplementary material for: How do phytophagous insects affect phyllosphere fungi? Tracking fungi from milkweed to monarch caterpillar frass reveals communities dominated by fungal yeast
Source: Environ Microbiol Rep. 2024 May 13;16(3):e13213. doi: 10.1111/1758-2229.13213 (PMC11089944; doi:10.1111/1758-2229.13213)
Supplement: Supplementary file 8 — APPENDIX S8. Cultured fungi from caterpillar frass immediately collected from caterpillars. [file EMI4-16-e13213-s003.pdf]

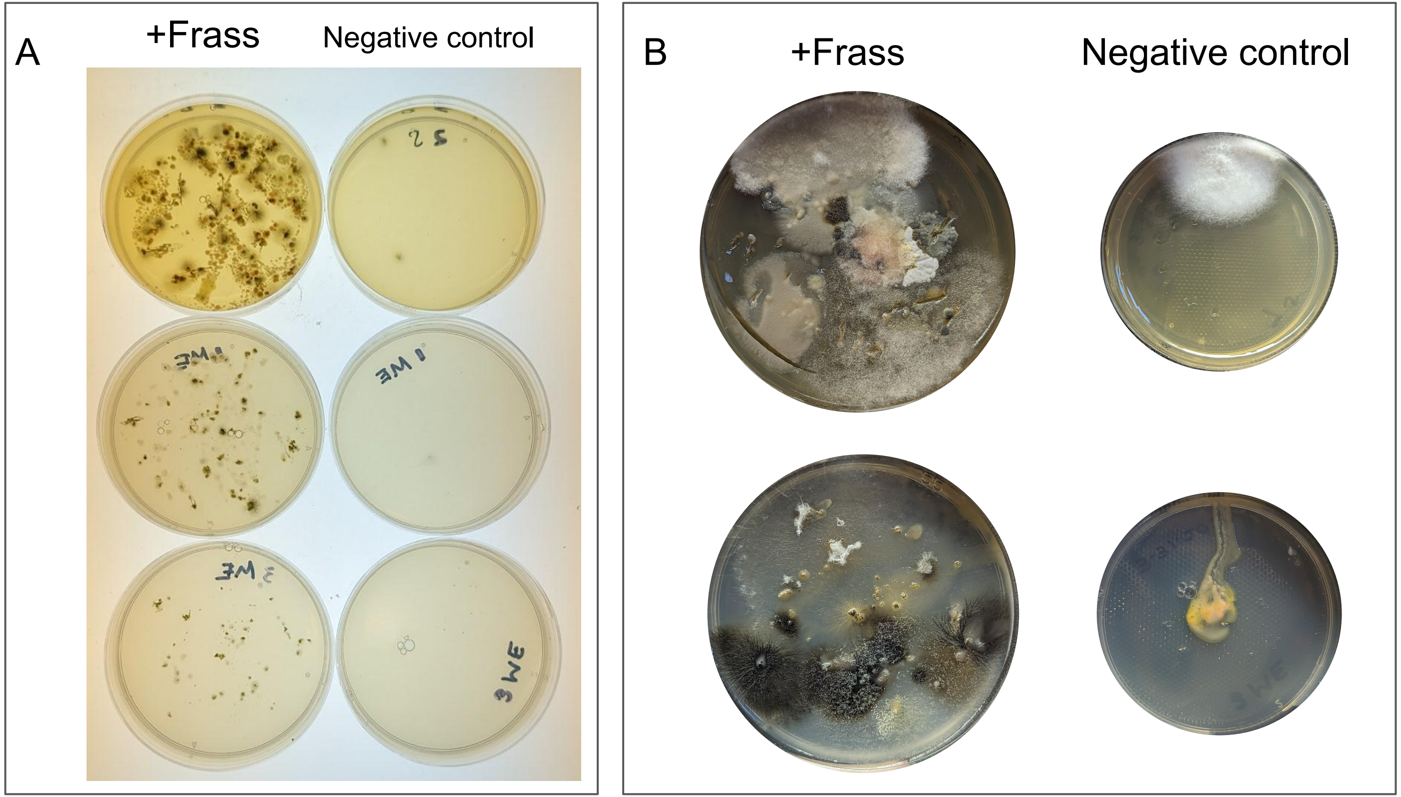

Appendix S8. Results of culturing frass immediately collected from monarch caterpillars. A. Pictures of plates 7 days after frass were plated on Sabouraud's media (top) or 2% malt extract (bottom two). B. Pictures of plates 3 weeks after frass were plated on Sabouraud's media (top) or 2% malt extract (bottom). Bottom two photos are the same two plates at the bottom in A.
